# Lyapunov function and global asymptotic stability for a new multiscale viral dynamics model incorporating the immune system response: Implemented upon HCV

**DOI:** 10.1371/journal.pone.0257975

**Published:** 2021-10-12

**Authors:** Hesham A. Elkaranshawy, Hossam M. Ezzat, Nermeen N. Ibrahim

**Affiliations:** 1 Faculty of Engineering, Department of Engineering Mathematics and Physics, Alexandria University, Alexandria, Egypt; 2 High Institute of Public Health, Alexandria University, Alexandria, Egypt; National Institute of Infectious Diseases, JAPAN

## Abstract

In this paper, a new mathematical model is formulated that describes the interaction between uninfected cells, infected cells, viruses, intracellular viral RNA, Cytotoxic T-lymphocytes (CTLs), and antibodies. Hence, the model contains certain biological relations that are thought to be key factors driving this interaction which allow us to obtain precise logical conclusions. Therefore, it improves our perception, that would otherwise not be possible, to comprehend the pathogenesis, to interpret clinical data, to control treatment, and to suggest new relations. This model can be used to study viral dynamics in patients for a wide range of infectious diseases like HIV, HPV, HBV, HCV, and Covid-19. Though, analysis of a new multiscale HCV model incorporating the immune system response is considered in detail, the analysis and results can be applied for all other viruses. The model utilizes a transformed multiscale model in the form of ordinary differential equations (ODE) and incorporates into it the interaction of the immune system. The role of CTLs and the role of antibody responses are investigated. The positivity of the solutions is proven, the basic reproduction number is obtained, and the equilibrium points are specified. The stability at the equilibrium points is analyzed based on the Lyapunov invariance principle. By using appropriate Lyapunov functions, the uninfected equilibrium point is proven to be globally asymptotically stable when the reproduction number is less than one and unstable otherwise. Global stability of the infected equilibrium points is considered, and it has been found that each equilibrium point has a specific domain of stability. Stability regions could be overlapped and a bistable equilibria could be found, which means the coexistence of two stable equilibrium points. Hence, the solution converges to one of them depending on the initial conditions.

## 1. Introduction

Hepatitis C virus (HCV) is a bloodborne virus that has become one of the most serious infectious diseases that threaten human health [1]. It can lead to both acute and chronic hepatitis, ranging in severity from a mild to a serious lifelong illness. A significant number of those who are chronically infected will develop cirrhosis or liver cancer, where HCV is a major cause of liver cancer. An estimated 71 million people have chronic HCV infections worldwide. The world health organization (WHO) estimated that in 2016, approximately 399 000 people died from hepatitis C, mostly from cirrhosis and liver cancer [2]. Treatment for chronic hepatitis C infection began in the early 1990s with interferon-alfa [3]. This injectable drug worked by improving the immune system, rather than by specifically attacking the virus. In 1998, the oral drug ribavirin was added to interferon [4]. The development of the treatment occurred in 2002 with the approval of pegylated interferon-alfa, a process that makes interferon more durable and effective [5]. New treatment options include direct-acting antiviral agents (DAAs) targeting specific HCV-life cycle components [6]. The quick steps of HCV drug development where a cure rate of more than 95% was achieved [2], have led to the hopeful prediction that full eradication of HCV is theoretically possible in the absence of a vaccine for HCV. There remain many barriers that need to be overcome. Further studies for factors that increase the eradication rate are needed. Such barriers include factors related to awareness, linkage to care, the development and availability of simplified and highly effective drug regimens, improving the rates of detection of infection, and the availability of funds expertise [7,8].

Mathematical modeling is a useful tool to study and analyze many engineering and physical problems. It has also been used to describe some biological processes such as heartbeats [9], tumor growth and cancer treatment [10,11], and virus dynamics for many types of viruses such as HCV, HBV, HIV, and Covid-19 [12–14]. It provides a powerful tool in the study of virus dynamics because it helps to understand the biological mechanisms and interpret the experimental results. Mathematical models can be used to predict the virus behavior under certain conditions or to determine which parameters increase disease spread. They can also be used to predict the number of medications required to help eradicate a disease or control it [9]. Mathematical modeling is also useful in public health policy formulation addressing the control of infectious diseases [15]. The early mathematical model for HCV was developed and analyzed in [16,17] as a scheme consisting of a system of ordinary differential equations describing the basic dynamics of the hepatitis C virus in-vivo. Models for HCV treatment with DAAs therapy is considered in [18–22]. A novel approximate analytical solution for solving the standard viral dynamic model for HCV is presented in [23]. Local and global stability analysis for basic virus dynamics models is studied in [24–26].

Interactions between replicating virus, liver cells, and different types of immune responses (CTLs and antibodies) are highly complex and nonlinear, so these interactions between HCV and the immune system were studied with mathematical models in [27,28] and stability has been analyzed for these models. However, these models can only describe the intercellular viral dynamics and cannot describe the intracellular viral dynamics which is required to capture the different antiviral effects corresponding to the action mechanisms of drugs.

Authors in [29–32] constructed a multiscale model that accounts for the dynamics of intracellular viral replication, and which includes the major stages in the HCV life cycle that are targeted by DAAs. These multiscale models have been developed using partial differential equations (PDEs). Since numerical PDE solvers are time-consuming and often converge poorly, a new approach has been suggested by Kitagawa, et al. [33] that converts a standard PDE multiscale model of the HCV infection into an equivalent system of ordinary differential equations (ODEs) without any assumptions. This transformed model prevents time-consuming calculations and has become widely available for further mathematical analysis. Kitagawa, et al. [34] derived the basic reproduction number of the transformed ODE model and studied the global stability of the model using Lyapunov–LaSalle’s invariance principle and investigated all possible steady states of the model. Local stability analysis of this model is considered in [35] using Routh-Hurwitz criterion. In that work, sensitivity analysis had been performed to specify the influence of each parameter on the basic reproduction number.

It is worth mentioning that the classical multiscale model ignores the responses of the immune system, which have a significant role in reducing viral load. From the point of view of the mathematical analysis, considering the immune system with a multiscale model in the PDE form is an undesirable task. In this paper, a new mathematical model, that deals with the interaction between the transformed multiscale model of the HCV infection in ODE form and immune responses, is proposed. Different antiviral effects of multidrug treatments are presented by defining three efficacies which are responsible for blocking intracellular viral production, blocking virion assembly/secretion, and enhancing the degradation rate of vRNA. The model contributes to improving our realization to the interactions between HCV, drug treatments, infected cells, and immune system. For instance, the analysis of the model reveals the existence of five equilibrium points: an uninfected point, an infected point with no immune responses, an infected point with dominant antibody responses without CTLs, an infected point with dominant CTLs responses without antibody, and an infected point with coexistence responses of both CTLs and antibody. In section 2, the ODE model extracted from the multiscale model for describing the dynamics of the HCV infection is described. Consequently, this model is extended to consider the impact of the immune system response. The proof of the positivity of the extended model and calculation of the reproduction number for the model is presented in section 3. The equilibrium points are determined in section 4 and the stability analysis is presented in section 5. Finally, section 6 concludes the paper.

## 2. Extended model for the transformed multiscale ODE HCV model

A multiscale model in the form of PDEs, which describes the intracellular life cycle had been proposed and applied by many researchers [29–32] for analyzing clinical data under multidrug treatment. The model is as follows:

$$\frac{\partial R(a,t)}{\partial t}+\frac{\partial R(a,t)}{\partial a}=\alpha (1-\varepsilon_{\alpha })-((1-\varepsilon_{s})\rho +k\mu )R(a)$$ (1)

$$\frac{dT(t)}{dt}=s-r\;T(t)-\beta \;V(t)T(t)$$ (2)

$$\frac{\partial i(a,t)}{\partial t}+\frac{\partial i(a,t)}{\partial a}=-\delta \;i(a,t)$$ (3)

$$\frac{dV(t)}{dt}=(1-\varepsilon_{s})\rho \int_{0}^{\infty }R(a,t)i(a,t)da-c\;V(t)$$ (4)

The variables *T*(*t*) and *V*(*t*) are the numbers of target cells and viruses, respectively, and the variable *i*(*a*,*t*) represents the age distribution of infected cells. Similarly, *R*(*a*,*t*) is the age and time distribution of intracellular viral RNA (vRNA) in a cell with infection age *a*. The target cells are assumed to be produced at rate *s*, infected by viruses at rate *β*, and naturally die at rate *r*. The infected cells die at rate *δ*, and virions are cleared at rate *c*. The parameters *α* and *μ* denote the production and degradation rates of the intracellular viral RNA, respectively. Viral RNA is assumed to assemble along with viral proteins and to be secreted from an infected cell as viral particles at rate *ρ*. The model recognizes the different antiviral effects of multidrug treatments by defining three efficacies *ε*_*α*_, *ε*_*s*_, and *k*≥1, which are responsible for the actions of blocking intracellular viral production, blocking virion assembly and/or secretion, and increasing the degradation rate of vRNA, respectively.

Kitagawa et al. [33] transformed the previous multiscale PDE model into the following ODEs model:

$$\frac{dT(t)}{dt}=s-r\;T(t)-\beta \;V(t)T(t)$$ (5)

$$\frac{dI(t)}{dt}=\beta \;V(t)T(t)-\delta \;I(t)$$ (6)

$$\frac{dP(t)}{dt}=\zeta \beta \;V(t)T(t)+\alpha (1-\varepsilon_{\alpha })I(t)-(k\mu +\rho (1-\varepsilon_{s})+\delta )P(t)$$ (7)

$$\frac{dV(t)}{dt}=\rho (1-\varepsilon_{s})P(t)-c\;V(t)$$ (8)

where *I*(*t*) denotes the total number of infected cells and is defined as $I(t)=\int_{0}^{\infty }i(a,t)da$, and *P*(*t*) is the total amount of intracellular viral RNA pooled in all infected cells and defined as $P(t)=\int_{0}^{\infty }R(a,t)i(a,t)da$. The entry virus-derived RNA starts to replicate from ζ copies in a newly infected cell and is fixed to 1 [33,34].

In this work, an extension to the transformed multiscale ODE model is proposed. Two more variables are added to stand for the immune system response. The first one represents the CTLs number which is responsible for killing the infected cells accordingly inhibiting the reproduction of the virus and is denoted by *Z*(*t*). The second one represents the number of the antibodies generated which is responsible for neutralizing the virus in-vivo and is denoted by *W*(*t*). Hence, the proposed model is described by the following ODEs system:

$$\frac{dT(t)}{dt}=s-r\;T(t)-\beta \;V(t)T(t)$$ (9)

$$\frac{dI(t)}{dt}=\beta \;V(t)T(t)-\delta \;I(t)-f\;I(t)Z(t)$$ (10)

$$\frac{dP(t)}{dt}=\beta \;V(t)T(t)+\alpha (1-\varepsilon_{\alpha })I(t)-(k\mu +(1-\varepsilon_{s})\rho +\delta )P(t)$$ (11)

$$\frac{dV(t)}{dt}=\rho (1-\varepsilon_{s})P(t)-c\;V(t)-q\;V(t)W(t)$$ (12)

$$\frac{dZ(t)}{dt}=u\;I(t)Z(t)-b\;Z(t)$$ (13)

$$\frac{dW(t)}{dt}=gV(t)W(t)-h\;W(t)$$ (14)

The term *f I*(*t*)*Z*(*t*) in Eq (10) represents the rate of killing the infected cells by the CTL response and the term *q V*(*t*)*W*(*t*) in Eq (12) represents the rate of neutralizing virus particles by the antibodies. CTLs become activated in response to viral antigen derived from infected cells, and once activated, they are divided, and their population grows (clonal expansion). So, in Eq (13), the CTLs increase at a rate of *uI*(*t*)*Z*(*t*). The CTLs decay at a rate of *bZ*(*t*) due to the lack of antigenic stimulation. Antibodies are produced by B cells and initially they are attached to them. They serve as the receptor that can specifically recognize the virus. When the B cells are exposed to a free virus, they divide and secrete the antibodies. Accordingly, antibodies progress at a rate *gV*(*t*)*W*(*t*) and decay at a rate *hW*(*t*) in Eq (14).

To avoid complexity in the mathematical analysis, the saturation effects of the concentrations of all variables are not contained in the proposed model. Yet, since the proliferation terms are not limited by saturation, the model could predict unlimited increases in the values of these variables which are certainly unrealistic. In general, however, the model can describe the dynamics of these variables and to gain important insights, as long as one is aware of the model limitations, and the results obtained do not depend on the unrealistic values of variables.

The proposed model presented by Eqs (9)–(14) can be used for numerical simulations to represent a variety of medical cases under treatment and can be a valuable tool to comprehend the pathogenesis and in controlling treatment of chronic HCV. Yet, the computation of the basic reproduction number, the determination of the equilibrium points, and stability analysis for this model have to be accomplished under no treatment. No treatment can be specified by assigning *ε*_*α*_ = 0, *ε*_*s*_ = 0, and *k* = 1. For clarity and effectiveness, we demonstrate the form of the proposed model under no treatment as:

$$\frac{dT(t)}{dt}=s-r\;T(t)-\beta \;V(t)T(t)$$ (15)

$$\frac{dI(t)}{dt}=\beta \;V(t)T(t)-\delta \;I(t)-f\;I(t)Z(t)$$ (16)

$$\frac{dP(t)}{dt}=\beta \;V(t)T(t)+\alpha \;I(t)-(\mu +\rho +\delta )P(t)$$ (17)

$$\frac{dV(t)}{dt}=\rho \;P(t)-c\;V(t)-q\;V(t)W(t)$$ (18)

$$\frac{dZ(t)}{dt}=u\;I(t)Z(t)-b\;Z(t)$$ (19)

$$\frac{dW(t)}{dt}=gV(t)W(t)-h\;W(t)$$ (20)

## 3. Basic properties of the extended model

### 3.1 Non-negativity of the solutions

To retain the biological fidelity of the model, the solutions to the mathematical model have to be non-negative.

**Theorem 3.1.** *Let τ>0. If the initial conditions satisfy T*(0)≥0, *I*(0)≥0, *P*(0)≥0, *V*(0)≥0, *Z*(0)≥0 and *W*(0)≥0 *then for all t*∈[0, *τ*], *T*(*t*), *I*(*t*), *P*(*t*), *V*(*t*), *Z*(*t*) *and W*(*t*) *will remain non-negative in* ℝ^6^.

*Proof*. We know that all of the parameters used in the system are positive. Thus, we can place lower bounds on each of the Eqs (9)–(14). Thus,

$$\frac{dT(t)}{dt}\geq -r\;T(t)-\beta \;V(t)T(t)$$

$$\frac{dI(t)}{dt}\geq -\delta \;I(t)-f\;I(t)Z(t)$$

$$\frac{dP(t)}{dt}\geq -(k\mu +(1-\varepsilon_{s})\rho +\delta )P(t)$$

$$\frac{dV(t)}{dt}\geq -c\;V(t)-q\;V(t)W(t)$$

$$\frac{dZ(t)}{dt}\geq -b\;Z(t)$$

$$\frac{dW(t)}{dt}\geq -h\;W(t)$$

Through basic differential equations methods, we can resolve the inequalities and produce:

$$T(t)\geq e^{-r\;t-\beta \;\int \;V(t)dt}\geq 0,$$

$$I(t)\geq e^{-\delta \;t-f\;\int \;Z(t)dt}\geq 0,$$

$$P(t)\geq e^{-(k\mu +(1-\varepsilon_{s})\rho +\delta )t}\geq 0,$$

$$V(t)\geq e^{-c\;t-q\;\int \;W(t)dt}\geq 0$$

$$Z(t)\geq e^{-b\;t}\geq 0,$$

$$W(t)\geq e^{-h\;t}\geq 0,$$

Thus, for all *t*∈[0,*τ*], *T*(*t*), *I*(*t*), *P*(*t*), *V*(*t*), *Z*(*t*) *and W*(*t*) will remain non-negative in ℝ^6^.

### 3.2 Computation of the basic reproduction number (R_0_)

The basic reproduction number is defined as the expected total number of viral particles newly produced during the whole period of infection from one typical viral particle in a population consisting only of uninfected cells. Accordingly, the basic reproduction number *R*_0_ is calculated under no treatment condition described by Eqs (15)–(20), and it is also computed at disease-free equilibrium *E*_0_. *E*_0_ is the uninfected equilibrium point, which will be explained in section 4, with *T* = *s*/*r*,*I* = *P* = *V* = *Z* = *W* = 0. It can be written as *E*_0_ = (*T*_0_ = *s*/*r*,0,0,0,0,0). This basic reproduction number explains the average number of newly infected cells based on the dynamics of the total amount of intracellular viral RNA, which corresponds to *P*(*t*) in the transformed ODE model, instead of the dynamics of the individual amount of intracellular viral RNA in the original PDE model. Note that the life cycles of both extracellular viral and total intracellular viral RNA are explicitly considered in the ODE model, and the viruses are formulated from the viral RNAs.

Many methods can be used to obtain the basic reproduction number, see for example [36]. The chosen method is the next-generation method, which was introduced Diekmann et. al., [37]. There are two principal approaches to apply this method elaborated by Driessche and Watmough [38] and by Castillo-Chavez, et. al., [39]. In this work, the second approach is considered, and an outline of this approach is given, proofs and further details can be found in [36,37,39]. Variables *T*(*t*), *I*(*t*), *P*(*t*), *V*(*t*), *Z*(*t*) and *W*(*t*) can be discretized into three groups: the non-infected group *ϕ*, the infected but not infectious group *ψ*, and the infected and infectious group *γ*. Hence, we have *ϕ* = (*T*,*Z*,*W*), *ψ* = (*I*,*P*), and *γ* = (*V*). The model in Eqs (15)–(20) can be written as

$$\frac{d\phi }{dx}=f(\phi ,\psi ,\gamma )$$ (21)

$$\frac{d\psi }{dx}=g(\phi ,\psi ,\gamma )$$ (22)

$$\frac{d\gamma }{dx}=h(\phi ,\psi ,\gamma )$$ (23)

The uninfected equilibrium point is given by *E*_0_ = (*ϕ*_0_,*ψ*_0_,*γ*_0_), where *ϕ*_0_ = (*T*_0_,0,0), *ψ*_0_ = (0,0), and *γ*_0_ = (0). Considering *g*(*ϕ*_0_,*ψ*,*γ*) = 0 gives:

$$\beta \;T_{0}\;V-\delta \;I=0$$ (24)

$$\beta \;T_{0}\;V+\alpha \;I-(\mu +\rho +\delta )P=0$$ (25)

Solving these two equations for *I* and *P* in terms of *V*, gives:

$$I=\frac{\beta \;T_{0}}{\delta }V$$ (26)

$$P=\frac{\beta \;T_{0}(\alpha +\delta )}{\delta (\mu +\rho +\delta )}V$$ (27)

Substituting in *h* (*ϕ*_0_,*I*,*P*,*V*) leads to:

$$h(\phi_{0},I(\phi_{0},V),P(\phi_{0},V),V)=\frac{\rho \;\beta \;T_{0}(\alpha +\delta )}{\delta (\mu +\rho +\delta )}V-c\;V$$ (28)

Let

$$G=\frac{d}{dV}h\;(\phi_{0},I(\phi_{0},0),P(\phi_{0},0),0)=\frac{\rho \;\beta \;T_{0}(\alpha +\delta )}{\delta (\mu +\rho +\delta )}-c$$ (29)

hence:

$$G=\frac{\rho \;\beta \;T_{0}(\alpha +\delta )}{\delta (\mu +\rho +\delta )}-c$$ (30)

*G* can be written as:

$$G=M-D,\;where\;M=\frac{\rho \beta \;T_{0}(\alpha +\delta )}{\delta (\mu +\rho +\delta )}and\;D=c$$ (31)

Hence, the basic reproductive number is the spectral radius:

$$R_{0}=\rho_{1}(MD^{-1})$$ (32)

that means that:

$$R_{0}=\frac{\beta \;s\;\rho (\alpha +\delta )}{c\;r\;\delta (\mu +\rho +\delta )}$$ (33)

## 4. The equilibrium points

Equilibrium points are the values of the variables T_*_, I_*_, P_*_, V_*_, Z_*_ and W_*_, under no treatment, at which the derivatives of these variables, i.e., the left-hand sides in Eqs (15)–(20), vanish. These equilibrium points represent the steady states after the cease of medication. In Fact, in stability analysis the interest is in specifying the behavior of the virus after the cease of medication. Hence, these equilibrium points satisfy the following algebraic equations:

$$s-r\;T_{*}-\beta \;V_{*}T_{*}=0$$ (34)

$$\beta \;V_{*}T_{*}-\delta \;I_{*}-f\;I_{*}Z_{*}=0$$ (35)

$$\beta \;V_{*}T_{*}+\alpha \;I_{*}-(\mu +\rho +\delta )P_{*}=0$$ (36)

$$\rho \;P_{*}-c\;V_{*}-q\;V_{*}W_{*}=0$$ (37)

$$u\;I_{*}Z_{*}-b\;Z_{*}=0$$ (38)

$$gV_{*}W_{*}-h\;W_{*}=0$$ (39)

The commercial program *Mathematica 12 program* is used solve these algebraic equations to obtain the equilibrium points. The program gives six equilibrium points, however, one of them has negative coordinates that have no biological meaning. The five other points are:

$$E_{0}=\left(\frac{s}{r},0,0,0,0,0\right)$$ (40)

$$E_{1}=(\frac{s}{rR_{0}},\frac{s}{\delta }\left(1-\frac{1}{R_{0}}\right),\frac{c\;r}{\beta \rho }(R_{0}-1),\frac{r}{\beta }(R_{0}-1),0,0)$$ (41)

$$E_{2}=(k_{2}s,\frac{\beta hS}{\delta g}k_{2},\frac{c\;r\;hR_{0}}{g\rho }k_{2},\frac{h}{g},0,\frac{c}{q}(-1+r\;k_{2}R_{0}))$$ (42)

$$E_{3}=(\frac{k_{1}+2c\;r\;u\;\mu_{1}-k_{3}}{2\beta \;r\;\rho u},\frac{b}{u},\frac{k_{1}+k_{3}}{2\beta \;\rho \;\mu_{1}u},\frac{k_{1}+k_{3}}{2\beta \;c\;\mu_{1}u},\frac{{(k}_{1}+k_{3})}{2\;b\;\beta \rho f}-\frac{\left(\alpha +\delta \right)}{f},0)$$ (43)

$$E_{4}=(k_{2}s,\frac{b}{u},\frac{c\delta \;r\;R_{0}}{s\rho (\alpha +\delta )}\left(\frac{\alpha \;b}{u\beta }+\frac{sk_{2}h}{g}\right),\frac{h}{g},\left(\frac{\beta \;h\;s\;u}{brgf+b\beta hf}-\frac{\delta }{f}\right),\left(\frac{\alpha \;bg\rho }{h\mu_{1}qu}+\frac{\beta k_{2}\rho s}{\mu_{1}q}-\frac{c}{q}\right))$$ (44)

where

$$\mu_{1}=(\delta +\mu +\rho )$$

$$k_{1}=\alpha \;b\;\beta \;\rho +\beta \rho \;s\;u-c\;r\;u\;\mu_{1}$$

$$k_{2}=\frac{g}{r\;g+\beta h}$$

$$k_{3}=\sqrt{4\alpha \;b\;\beta \;c\;r\;\rho \mu_{1}u+{k_{1}}^{2}}$$

The first point, *E*_0_, is a virus-free equilibrium point, while the other four points are virus-infected. These four infected equilibrium points are: an infected state with no immune responses, an infected state with dominant antibody responses without CTLs, an infected state with dominant CTL responses without antibodies, and an infected state with coexistence responses of both CTLs and antibodies, respectively.

Since the equilibrium points should have non-negative coordinates only, the following conditions for existence can be obtained:

### Remark 4.1

1. *E*_1_ exists only if *R*_0_≥1. When *R*_0_ = 1 then *E*_1_ = *E*_0_.
2. *E*_2_ exists only if *R*_0_≥*A*_1_. When *R*_0_ = *A*_1_ then *E*_2_ = *E*_1_.
3. *E*_3_ exists only if $\frac{{(k}_{1}+k_{3})}{2\;b\;\beta \rho f}\geq \frac{\left(\alpha +\delta \right)}{f}$, which by simplification leads to *R*_0_≥*A*_2_. It is also required that 2 *c r u* μ_1_≥*k*_3_−*k*_1_, however, this is always true and would not add a new condition. When *R*_0_ = *A*_2_ then *E*_3_ = *E*_1_.
4. *E*_4_ exists only if *R*_0_≥*A*_4_ and *R*_0_≥*A*_3_. When *R*_0_ = *A*_3_ then *E*_4_ = *E*_2_ and when *R*_0_ = *A*_4_ then *E*_4_ = *E*_3_.

Where, $A_{1}=1+\frac{\beta \;h}{r\;g},A_{2}=1+\frac{\beta \;\rho \;b(\alpha +\delta )}{c\;r\;u\;\mu_{1}},A_{3}=\frac{g\rho \;b(\alpha +\delta )}{c\;r\;u\;h\;k_{2}\mu_{1}}$, and $A_{4}=\frac{\beta \;s\;u\;h(\alpha +\delta )}{r\;\delta (\alpha \;b\;g+\beta \;s\;u\;h\;k_{2})}$

These conditions are necessary and sufficient conditions for the existence and non-existence of the equilibrium points.

## 5. Global stability analysis

Usually, the global stability analysis of a dynamical system is a very complex problem. One of the most efficient methods to solve this problem is Lyapunov’s theory. To build the Lyapunov function, the technique used in [24–26,40], which had been suggested and utilized for other models, is adopted. In this section, the global asymptotic stability of the model for both the uninfected and the infected equilibrium points is investigated.

Assume the following general form of Lyapunov function *l*(*t*):

$$l(t)=\epsilon_{1}\left(T-T_{*}-T_{*}\ln\left(\frac{T}{T_{*}}\right)\right)+\epsilon_{2}\left(I-I_{*}-I_{*}\ln\left(\frac{I}{I_{*}}\right)\right)+\epsilon_{3}(P-P_{*}-P_{*}\ln\left(\frac{P}{P_{*}}\right))+\epsilon_{4}\left(V-V_{*}-V_{*}\ln\left(\frac{V}{V_{*}}\right)\right)+\epsilon_{5}(Z-Z_{*}-Z_{*}\ln\left(\frac{Z}{Z_{*}}\right))+\epsilon_{6}\left(W-W_{*}-W_{*}\ln\left(\frac{W}{W_{*}}\right)\right)$$ (45)

where T_*_ means the value of *T* at the equilibrium point, and whenever T_*_ is zero the corresponding *ln* term does not exist, and this is applied to all other variables. ϵ_1_, ϵ_2_, ϵ_3_, ϵ_4_, ϵ_5_, and ϵ_6_ are constants and will be specified through the proof of the stability of equilibrium points.

### 5.1 Basic properties of Lyapunov function *l*(*t*)

The following properties should be demonstrated in any Lyapunov function *l*(*t*):

1. It is a continuously differentiable function defined in domain *D*⊂ℝ^6^; 0∈*D*, and defined for all *T*(*t*)≥0, *I*(*t*)≥0, *P*(*t*)≥0, *V*(*t*)≥0, *Z*(*t*)≥0 and *W*(*t*)≥0. This property is already satisfied in the proposed function in Eq (45).
2. It is always nonnegative function in ℝ^6^, but equal to 0 at the equilibrium points. In the following subsections, the conditions required for the equilibrium points to fulfill this property are obtained.
3. It satisfies the radial unboundedness condition i.e., if any dependent variable tends to infinity, *l*(*t*) also tends to infinity. To show this, let us assume that *T*(*t*) in Eq (45) tends to infinity, then:

$$l(t)=\epsilon_{1}\underset{T\to \infty }{\lim}(T-T_{*}-T_{*}\ln\left(\frac{T}{T_{*}}\right))+Finite\;terms=\epsilon_{1}\underset{T\to \infty }{\lim}T(1-\frac{T_{*}}{T}-\frac{\ln\left(T/T_{*}\right)}{T/T_{*}})+Finite\;terms$$

Using L’Hôpital’s rule, $\frac{\ln\left(T/T_{*}\right)}{T/T_{*}}$ tends to zero, hence, *l*(*t*) tends to infinity.

### 5.2 Global stability of uninfected equilibrium point

**Theorem 5.1.** *E*_0_ is globally asymptotically stable if *R*_0_≤1.

***Proof*:** Consider the following Lyapunov function for the uninfected equilibrium point *E*_0_(*T*_0_,0,0,0,0,0):

$$l(t)=\left(1+\frac{\alpha }{\delta }\right)\left(T-T_{0}-T_{0}\ln\left(\frac{T}{T_{*}}\right)\right)+\frac{\alpha }{\delta }I+P+\frac{\mu_{1}}{\rho }V+\frac{\alpha }{\delta }\frac{f}{u}Z+q\frac{\mu_{1}}{\rho \;g}W$$

In this case $\epsilon_{1}=(1+\frac{\alpha }{\delta }),\epsilon_{2}=\frac{\alpha }{\delta },\epsilon_{3}=1,\epsilon_{4}=\frac{\mu_{1}}{\rho },\epsilon_{5}=\frac{\alpha }{\delta }\frac{f}{u},\epsilon_{6}=q\frac{\mu_{1}}{\rho \;g}$. The time derivative of *l*(*t*) is:

$$\frac{dl(t)}{dt}=(1+\frac{\alpha }{\delta })(1-\frac{T_{0}}{T})(s-r\;T-\beta \;V\;T)+(\frac{\alpha }{\delta })(\beta \;V\;T-\delta \;I-f\;I\;Z)+(\beta \;V\;T+\alpha I-\mu_{1}P)+(\frac{\mu_{1}}{\rho })(\rho P-c\;V-q\;V\;W)+(\frac{\alpha }{\delta }\frac{f}{u})(u\;I\;Z-b\;Z)+q\frac{\mu_{1}}{\rho g}(g\;V\;W-h\;W)$$

Eq (45) can be simplified by substituting *s* = *r T*_0_, which leads to:

$$\frac{dl(t)}{dt}=\left(1+\frac{\alpha }{\delta }\right)(T-T_{0})\left(r\frac{T_{0}}{T}-r-\beta \;V\right)+\frac{\alpha }{\delta }(\beta \;V\;T)+\frac{\mu_{1}}{\rho }(-cV)+\frac{\alpha }{\delta }\frac{f}{u}(-b\;Z)+q\frac{\mu_{1}}{\rho g}(-h\;W)$$

$$\frac{dl(t)}{dt}=\left(1+\frac{\alpha }{\delta }\right)(T-T_{0})\left(r\frac{T_{0}}{T}-r\right)+\left(1+\frac{\alpha }{\delta }\right)\beta \;T_{0}V+\frac{\mu_{1}}{\rho }(-c\;V)+\frac{\alpha }{\delta }\frac{f}{u}(-b\;Z)+q\frac{\mu_{1}}{\rho g}(-h\;W)$$

$$\frac{dl(t)}{dt}=r\;T_{0}\left(1+\frac{\alpha }{\delta }\right)\left(2-\frac{T_{0}}{T}-\frac{T}{T_{0}}\right)-\frac{\alpha }{\delta }\frac{f}{u}b\;Z-q\frac{\mu_{1}}{\rho g}h\;W+\left((1+\frac{\alpha }{\delta })\beta T_{0}-c\frac{\mu_{1}}{\rho }\right)V$$

Since $R_{0}=\frac{\beta \;s\rho (\alpha +\delta )}{c\;r\;\delta \mu_{1}}$, then $\frac{dl(t)}{dt}$ can be simplified to

$$\frac{dl(t)}{dt}=r\;T_{0}\left(1+\frac{\alpha }{\delta }\right)\left(2-\frac{T_{0}}{T}-\frac{T}{T_{0}}\right)-\frac{\alpha }{\delta }\frac{f}{u}b\;Z-q\frac{\mu_{1}}{\rho g}h\;W-\frac{c\mu_{1}}{\rho }(1-R_{0})V$$

Since the arithmetical mean is greater than the geometrical mean, then $\left(2-\frac{T}{T_{0}}-\frac{T_{0}}{T}\right)<0$. Consequently, $\frac{dl(t)}{dt}<0$ for any coordinate values (*T*,*I*,*P*,*V*,*Z*,*W*) and $\frac{dl(t)}{dt}=0$ at the coordinates of the uninfected equilibrium point (*T*_0_,0,0,0,0,0). Therefore, *R*_0_≤1 ensures that $\frac{dl(t)}{dt}\leq 0$ which verifies that all the trajectories of the model converge to *E*_0_, that is, the uninfected equilibrium point *E*_0_ is globally asymptotically stable when *R*_0_≤1.

**Theorem 5**.**2**

1. *E_1_ is globally asymptotically stable if 1≤R_0_≤min(A_1_, A_2_)*.
2. *E*_2_ is globally asymptotically stable if *A*_1_≤*R*_0_≤*A*_3_.
3. *E*_3_ is globally asymptotically stable if *A*_2_≤*R*_0_≤*A*_4_.
4. *E*_4_ is globally asymptotically stable if *R*_0_≥*max*(*A*_3_, *A*_4_).

*Proof*:

Consider the Lyapunov function for the infected equilibrium points:

$$l(t)=\left(1+\frac{\alpha I_{*}}{\beta V_{*}T_{*}}\right)\left(T-T_{*}-T_{*}\ln\left(\frac{T}{T_{*}}\right)\right)+\frac{\alpha I_{*}}{\beta V_{*}T_{*}}\left(I-I_{*}-I_{*}\ln\left(\frac{I}{I_{*}}\right)\right)+(P-P_{*}-P_{*}\ln\left(\frac{P}{P_{*}}\right))+\frac{\mu_{1}}{\rho }\left(V-V_{*}-V_{*}\ln\left(\frac{V}{V_{*}}\right)\right)+\frac{f\alpha I_{*}}{u\;\beta V_{*}T_{*}}(Z-Z_{*}-Z_{*}\ln\left(\frac{Z}{Z_{*}}\right))+q\frac{\mu_{1}}{\rho \;g}\left(W-W_{*}-W_{*}\ln\left(\frac{W}{W_{*}}\right)\right)$$

The time derivative of *l*(*t*) is:

$$\frac{dl(t)}{dt}=(1+\frac{\alpha I_{*}}{\beta V_{*}T_{*}})(1-\frac{T_{*}}{T})(s-r\;T-\beta \;V\;T)+(\frac{\alpha I_{*}}{\beta V_{*}T_{*}})(1-\frac{I_{*}}{I})(\beta \;V\;T-\delta \;I-f\;I\;Z)+(1-\frac{P_{*}}{P})(\beta \;V\;T+\alpha \;I-\mu_{1}P)+(\frac{\mu_{1}}{\rho })(1-\frac{V_{*}}{V})(\rho P-c\;V-q\;V\;W)+(\frac{f\alpha I_{*}}{u\beta V_{*}T_{*}})(1-\frac{Z_{*}}{Z})(u\;I\;Z-b\;Z)+q\frac{\mu_{1}}{\rho g}(1-\frac{W_{*}}{W})(gV\;W-h\;W)$$ (46)

For the infected equilibrium points, Eq (46) can be simplified by the following substitutions from Eqs (15)–(20):

$$s=r\;T_{*}+\beta \;V_{*}T_{*},$$ (47)

$$\delta =\frac{\beta \;V_{*}T_{*}}{I_{*}}-fZ_{*},$$ (48)

$$\mu_{1}=\frac{\left(\alpha I_{*}+\beta V_{*}T_{*}\right)}{P_{*}},$$ (49)

$$c=\frac{\rho P_{*}-qV_{*}W_{*}}{V_{*}}$$ (50)

The six terms of Eq (46) can be simplified as shown:

The first term:

By using Eq (47), the first term can be simplified as shown:

$$\left(1+\frac{\alpha I_{*}}{\beta V_{*}T_{*}})(1-\frac{T_{*}}{T})(s-r\;T(t)-\beta \;V\;T)=(1+\frac{\alpha I_{*}}{\beta \;V_{*}T_{*}})(1-\frac{T_{*}}{T})(r\;T_{*}+\beta \;V_{*}T_{*}-r\;T-\beta \;V\;T)=(1+\frac{\alpha I_{*}}{\beta \;V_{*}T_{*}})r\;T_{*}(2-\frac{T}{T_{*}}-\frac{T_{*}}{T})+\beta \;V_{*}T_{*}(1-\frac{VT}{V_{*}T_{*}}-\frac{T_{*}}{T}+\frac{V}{V_{*}})+\frac{\alpha I_{*}}{\beta \;V_{*}T_{*}}\beta \;V_{*}T_{*}(1-\frac{VT}{V_{*}T_{*}}-\frac{T_{*}}{T}+\frac{V}{V_{*}}\right)$$

The second term:

By using Eq (48), the second term can be simplified as shown:

$$\left(\frac{\alpha I_{*}}{\beta V_{*}T_{*}})(1-\frac{I_{*}}{I})(\beta \;V\;T-\delta \;I-f\;I\;Z)=(\frac{\alpha I_{*}}{\beta \;V_{*}T_{*}})(1-\frac{I_{*}}{I})(\beta \;V\;T-\frac{\beta V_{*}T_{*}I}{I_{*}}+fZ_{*}I-f\;I\;Z)=(\frac{\alpha I_{*}}{\beta V_{*}T_{*}})\beta V_{*}T_{*}(\frac{VT}{V_{*}T_{*}}-\frac{I}{I_{*}}-\frac{I_{*}VT}{V_{*}T_{*}I}+1)+(\frac{\alpha I_{*}}{\beta V_{*}T_{*}})fZ_{*}I_{*}(\frac{I}{I_{*}}-\frac{I\;Z}{Z_{*}I_{*}}-1+\frac{Z}{Z_{*}}\right)$$

The third term:

By using Eq (49), the third term can be simplified as shown:

$$\left(1-\frac{P_{*}}{P})(\beta \;V\;T+\alpha I-(\mu_{1})P)=(1-\frac{P_{*}}{P})(\beta \;V\;T+\frac{\alpha }{\delta }\frac{\beta \;V_{*}T_{*}I}{I_{*}}-\frac{\alpha }{\delta }fZ_{*}I-(\alpha I_{*}+\beta \;V_{*}T_{*})\frac{P}{P_{*}})=(1-\frac{P_{*}}{P})(\beta \;V_{*}T_{*}(\frac{VT}{V_{*}T_{*}}-\frac{P}{P_{*}})+\frac{\alpha }{\delta }\beta \;V_{*}T_{*}(\frac{I}{I_{*}}-\frac{P}{P_{*}})+fZ_{*}I_{*}\frac{\alpha }{\delta }(\frac{P}{P_{*}}-\frac{I}{I_{*}}))=\beta \;V_{*}T_{*}(\frac{VT}{V_{*}T_{*}}-\frac{P}{P_{*}}-\frac{VTP_{*}}{V_{*}T_{*}P(t)}+1)+\frac{\alpha }{\delta }\beta \;V_{*}T_{*}(\frac{I}{I_{*}}-\frac{P}{P_{*}}-\frac{IP_{*}}{I_{*}P}+1)+fZ_{*}I_{*}\frac{\alpha }{\delta }(\frac{P}{P_{*}}-\frac{I}{I_{*}}-1+\frac{IP_{*}}{I_{*}P}\right)$$

The fourth term:

By using Eq (50), the fourth term can be simplified as shown:

$$\left(\frac{\mu_{1}}{\rho })(1-\frac{V_{*}}{V})(\rho P-cV-qVW)=(\frac{\mu_{1}}{\rho })(1-\frac{V_{*}}{V})(\rho P-V(\frac{\rho P_{*}-qV_{*}W_{*}}{V_{*}})-qVW)=(\frac{\mu_{1}}{\rho })(1-\frac{V_{*}}{V})(\frac{\rho }{\left(\mu_{1}\right)}(\mu_{1})P_{*}(\frac{P}{P_{*}}-\frac{V}{V_{*}})+qVW_{*}-qVW)=(\frac{\mu_{1}}{\rho })(1-\frac{V_{*}}{V})(\frac{\rho }{\left(\mu_{1}\right)}(\mu_{1})P_{*}(\frac{P}{P_{*}}-\frac{V}{V_{*}}))+(\frac{\mu_{1}}{\rho })(1-\frac{V_{*}}{V})(qVW_{*}-qVW)=(\beta \;V_{*}T_{*}+\alpha I_{*})(1-\frac{V_{*}}{V})(\frac{P}{P_{*}}-\frac{V}{V_{*}})+(\frac{\mu_{1}}{\rho })(1-\frac{V_{*}}{V})(qVW_{*}-qVW)=(\beta V_{*}T_{*}+{\frac{\alpha }{\delta }\delta I}_{*})(\frac{P}{P_{*}}-\frac{V}{V_{*}}-\frac{V_{*}P}{P_{*}V}+1)+(\frac{\mu_{1}}{\rho })(1-\frac{V_{*}}{V})(qVW_{*}-qVW)=(\beta V_{*}T_{*}+\frac{\alpha }{\delta }(\beta \;V_{*}T_{*}-fZ_{*}I_{*}))(\frac{P}{P_{*}}-\frac{V}{V_{*}}-\frac{V_{*}P}{P_{*}V}+1)+(\frac{\mu_{1}}{\rho })(1-\frac{V_{*}}{V})(qVW_{*}-qVW)=\beta \;V_{*}T_{*}(\frac{P}{P_{*}}-\frac{V}{V_{*}}-\frac{V_{*}P}{P_{*}V}+1)+\frac{\alpha }{\delta }(\beta \;V_{*}T_{*})(\frac{P}{P_{*}}-\frac{V}{V_{*}}-\frac{V_{*}P}{P_{*}V}+1)-\frac{\alpha }{\delta }(fZ_{*}I_{*})(\frac{P}{P_{*}}-\frac{V}{V_{*}}-\frac{V_{*}P}{P_{*}V}+1)+(\frac{\mu_{1}}{\rho })q(VW_{*}-VW-V_{*}W_{*}+V_{*}W\right)$$

The fifth term:

$$\left(\frac{f\;\alpha I_{*}}{u\;\beta V_{*}T_{*}}\right)(u\;I\;Z-b\;Z-Z_{*}u\;I+bZ_{*})$$

The sixth term:

$$q\frac{\mu_{1}}{\rho g}(gVW-h\;W-gVW_{*}+hW_{*})$$

Substituting the sixth terms in Eq (46) gives:

$$\frac{dl(t)}{dt}=(1+\frac{\alpha I_{*}}{\beta \;V_{*}T_{*}})r\;T_{*}(2-\frac{T}{T_{*}}-\frac{T_{*}}{T})+\beta \;V_{*}T_{*}(2-\frac{T_{*}}{T}+\frac{V}{V_{*}}-\frac{VTP_{*}}{V_{*}T_{*}P(t)})+\alpha I_{*}(2-\frac{I}{I_{*}}-\frac{I_{*}VT}{V_{*}T_{*}I}-\frac{T_{*}}{T}+\frac{V}{V_{*}})+(\frac{\alpha I_{*}}{\beta \;V_{*}T_{*}})fZ_{*}I_{*}(-1+\frac{Z}{Z_{*}})+\frac{\alpha }{\delta }\beta V_{*}T_{*}(2+\frac{I}{I_{*}}-\frac{IP_{*}}{I_{*}P}-\frac{V}{V_{*}}-\frac{V_{*}P}{P_{*}V})+\beta V_{*}T_{*}(-\frac{V}{V_{*}}-\frac{V_{*}P}{P_{*}V}+1)+\frac{\alpha }{\delta }(fZ_{*}I_{*})(\frac{V}{V_{*}}+\frac{V_{*}P}{P_{*}V}-\frac{I}{I_{*}}+\frac{IP_{*}}{I_{*}P}-2)+(\frac{\mu_{1}}{\rho })q(-V_{*}W_{*}+V_{*}W)+(\frac{\alpha I_{*}}{\beta V_{*}T_{*}}\frac{f}{u})(-bZ+bZ_{*})+q\frac{\mu_{1}}{\rho g}(-h\;W+hW_{*})$$

Substituting Eq (48) into the previous equation gives

$$\frac{dl(t)}{dt}=r\;T_{*}(1+\frac{\alpha I_{*}}{\beta \;V_{*}T_{*}})(2-\frac{T}{T_{*}}-\frac{T_{*}}{T})+\beta V_{*}T_{*}(3-\frac{T_{*}}{T}-\frac{V\;TP_{*}}{V_{*}T_{*}P(t)}-\frac{V_{*}P}{P_{*}V})+(\frac{\alpha I_{*}}{\beta V_{*}T_{*}})fI_{*}(-Z_{*}+Z)+\alpha I_{*}(4-\frac{IP_{*}}{I_{*}P}-\frac{V_{*}P}{P_{*}V}-\frac{I_{*}VT}{V_{*}T_{*}I}-\frac{T_{*}}{T})+(\frac{\mu_{1}}{\rho })q(-V_{*}W_{*}+V_{*}W)+(\frac{\alpha I_{*}}{\beta V_{*}T_{*}}\frac{f}{u})(-b\;Z+bZ_{*})+q\frac{\mu_{1}}{\rho \;g}(-hW+hW_{*})$$ (51)

Since the arithmetical mean is greater than the geometrical mean, i.e., the terms $\left(2-\frac{T}{T_{*}}-\frac{T_{*}}{T}),(3-\frac{T_{*}}{T}-\frac{V_{*}P}{P_{*}V}-\frac{VTP_{*}}{V_{*}T_{*}P}\right)$, and $\left(4-\frac{T_{*}}{T}-\frac{I_{*}VT}{V_{*}T_{*}I}-\frac{IP_{*}}{I_{*}P}-\frac{V_{*}P}{P_{*}V}\right)$ are negative.

For the infected equilibrium point *E*_1_: Substituting Z_*_ = 0 in Eq (48) and then substituting the result combined with Z_*_ = 0 and W_*_ = 0 into Eq (51) gives

$$\frac{dl(t)}{dt}=rT_{*}(1+\frac{\alpha \;I_{*}}{\beta \;V_{*}T_{*}})(2-\frac{T}{T_{*}}-\frac{T_{*}}{T})+\beta \;V_{*}T_{*}(3-\frac{T_{*}}{T}-\frac{V_{*}P}{P_{*}V}-\frac{VTP_{*}}{V_{*}T_{*}P})+\alpha I_{*}(4-\frac{IP_{*}}{I_{*}P}-\frac{V_{*}P}{P_{*}V}-\frac{I_{*}VT}{V_{*}T_{*}I}-\frac{T_{*}}{T})+\frac{\alpha }{\delta }f\;Z(I_{*}-\frac{b}{u})+q\frac{\mu_{1}}{\rho }W(V_{*}-\frac{h}{g})$$

As per our derivation, $\frac{dl(t)}{dt}=0$ only at the equilibrium point *E*_1_. Furthermore, for $I_{*}\leq \frac{b}{u}$ and $V_{*}\leq \frac{h}{g}$ it follows that $\frac{dl(t)}{dt}<0$. Substituting the coordinates of the equilibrium point leads to $R_{0}\leq \frac{u\;s}{u\;s-\delta \;b}$ and $R_{0}\leq 1+\frac{\beta \;h}{r\;g}$. Hence, according to Lyapunov–LaSalle’s invariant principle combined with remark 4.1, *E*_1_ exists and it is globally asymptotically stable if 1≤*R*_0_≤*min*(*A*_1_, *A*_2_).

For the infected equilibrium point *E*_2_: Substituting Z_*_ = 0 in Eq (48) and then substituting the result combined with Z_*_ = 0 and $V_{*}=\frac{g}{h}$ into Eq (51) gives:

$$\frac{dl(t)}{dt}=r\;T_{*}(1+\frac{\alpha I_{*}}{\beta \;V_{*}T_{*}})(2-\frac{T}{T_{*}}-\frac{T_{*}}{T})+\beta \;V_{*}T_{*}(3-\frac{T_{*}}{T}-\frac{V_{*}P}{P_{*}V}-\frac{VT\;P_{*}}{V_{*}T_{*}P})+\alpha I_{*}(4-\frac{IP_{*}}{I_{*}P}-\frac{V_{*}P}{P_{*}V}-\frac{I_{*}VT}{V_{*}T_{*}I}-\frac{T_{*}}{T})+\frac{\alpha }{\delta }f\;Z(I_{*}-\frac{b}{u})$$

$\frac{dl(t)}{dt}=0$ only at the equilibrium point *E*_2_. For $I_{*}\leq \frac{b}{u}$ it follows that $\frac{dl(t)}{dt}<0$. Substituting the coordinates of this equilibrium point leads to $R_{0}\leq \frac{g\rho \;b(\alpha +\delta )}{c\;r\;u\;hk_{2}\mu_{1}}$. Hence, according to Lyapunov–LaSalle’s invariance principle combined with remark 4.1, *E*_2_ exists and it is globally asymptotically stable if *A*_2_≤*R*_0_≤*A*_3_.

For the infected equilibrium point *E*_3_: Substituting W_*_ = 0, and $I_{*}=\frac{b}{u}$ into Eq (51) gives

$$\frac{dl(t)}{dt}=r\;T_{*}(1+\frac{\alpha I_{*}}{\beta V_{*}T_{*}})(2-\frac{T}{T_{*}}-\frac{T_{*}}{T})+\beta \;V_{*}T_{*}(3-\frac{T_{*}}{T}-\frac{V_{*}P}{P_{*}V}-\frac{VT\;P_{*}}{V_{*}T_{*}P})+\alpha I_{*}(4-\frac{IP_{*}}{I_{*}P}-\frac{V_{*}P}{P_{*}V}-\frac{I_{*}VT}{V_{*}T_{*}I}-\frac{T_{*}}{T})+q\frac{\mu_{1}}{\rho }W(V_{*}-\frac{h}{g})$$

It can be noticed that $\frac{dl(t)}{dt}=0$ only at the equilibrium point *E*_3_. For $V_{*}\leq \frac{h}{g}$ it follows that $\frac{dl(t)}{dt}<0$. Substituting the coordinates of this equilibrium point gives $\frac{k_{1}+k_{3}}{2\beta \;c\;\mu_{1}u}\leq \frac{h}{g}$, which by simplification leads to $R_{0}\leq \frac{\beta \;s\;u\;h\;(\alpha +\delta )}{r\;\delta \;(\alpha \;b\;g+\beta \;s\;u\;h\;k_{2})}$. Hence, according to Lyapunov–LaSalle’s invariant principle combined with remark 4.1, *E*_3_ exists and it is globally asymptotically stable if *A*_1_≤*R*_0_≤*A*_4_.

For the infected equilibrium point *E*_4_: Substituting $I_{*}=\frac{b}{u}$, and $V_{*}=\frac{g}{h}$ into Eq (51) gives

$$\frac{dl(t)}{dt}=r\;T_{*}(1+\frac{\alpha I_{*}}{\beta V_{*}T_{*}})(2-\frac{T}{T_{*}}-\frac{T_{*}}{T})+\beta \;V_{*}T_{*}(3-\frac{T_{*}}{T}-\frac{V_{*}P}{P_{*}V}-\frac{VT\;P_{*}}{V_{*}T_{*}P})+\alpha I_{*}(4-\frac{IP_{*}}{I_{*}P}-\frac{V_{*}P}{P_{*}V}-\frac{I_{*}V\;T}{V_{*}T_{*}I}-\frac{T_{*}}{T})$$

Only at the equilibrium point *E*_*4*_ the derivative $\frac{dl(t)}{dt}=0$ while $\frac{dl(t)}{dt}<0$ at any other point. According to Lyapunov–LaSalle’s invariance principle combined with remark 4.1, *E*_4_ exists and it is globally asymptotically stable if *R*_0_≥*max*(*A*_3_, *A*_4_). This ends the prove of theorem 5.2.

## 6. Simulations

In this section, the proposed model and the transformed model are numerically simulated. For each of them, the corresponding system of ODE’s is numerically solved using *Mathematica 12 program*. The parameter values, which were estimated from clinical datasets in [33], are summarized in (Table 1), and the immune system parameters, which were proposed in [41], are given in (Table 2). These parameters are used in the simulations. Some of the values of the parameters are not mentioned in the Tables 1 and 2 and the used values will be mentioned explicitly.

**Table 1:** Multiscale model parameter values.

Parameters	Parameter definition	Values and units
*s*	Production rate of target cells	cells/ml. day^−1^
*β*	The rate at which virus *V*(*t*) infects target cells *T*(*t*)	5×10^−8^ ml day−1virion^−1^
*r*	Death rate of target cells	0.01 day^−1^
*δ*	Natural death rate of infected cell	0.14 day^−1^
*α*	The age-dependent rates of vRNA production	40 day^−1^
*k*	Efficacy responsible for the action of secretion and increasing degradation rate of vRNA	1 day^−1^
μ	The age-dependent rates of vRNA degradation	1 day^−1^
c	Natural clearance rate of virus *V*(*t*)	day^−1^
*ρ*	The age-dependent rates of vRNA assembly/secretion	8.18 day^−1^
*ε*_*α*_	Efficacy responsible for the actions of blocking intracellular viral production	0.99
*ε*_*s*_	Efficacy responsible for the actions of blocking virion assembly	0.56

**Table 2:** Immune system parameter values.

Parameters	Parameter definition	Values
*u*	Expand rate of CTLs *Z*(*t*) in response to virus antigen derived from infected cells *I*(*t*)	4.4×10^−7^ day^−1^
*b*	Natural decay rate of CTLs *Z*(*t*) in the absence of antigenic stimulation	10^−2^day^−1^
*g*	Development rate of antibody *W*(*t*) in response to virus *V*(*t*)	10^−5^day^−1^
*h*	Natural decay rate of antibody *W*(*t*)	10^−2^day^−1^
*f*	The rate at which CTLs *Z*(*t*) kills infected cells *I*(*t*)	6.4×10^−4^ day^−1^
*q*	The rate at which antibody *W*(*t*) neutralized the virus *V*(*t*)	2 day^−1^

**Fig 1:**
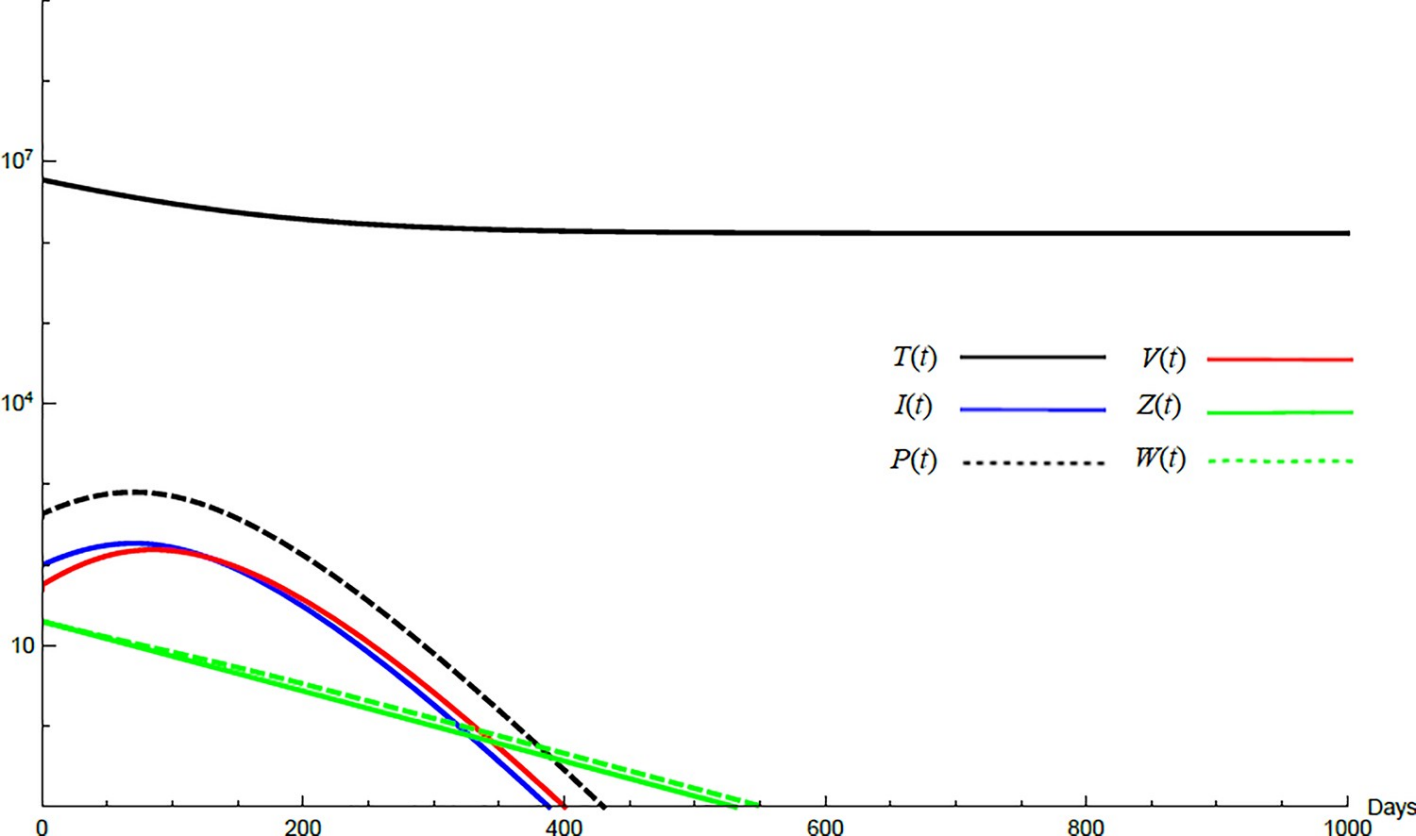
Variation of the variables for no treatment case with *s* = 1.3×10^4^, *c* = 22.3, *R*_0_ = 0.733. Hence *R*_0_<1 and *E*_0_ is stable. *E*_0_ = {1.3×10^6^,0,0,0,0,0}. *T*^0^ = 0.6×10^7^, *I*^0^ = 100, *P*^0^ = 400, *V*^0^ = 50, *Z*^0^ = 20, *W*^0^ = 20.

**Fig 2:**
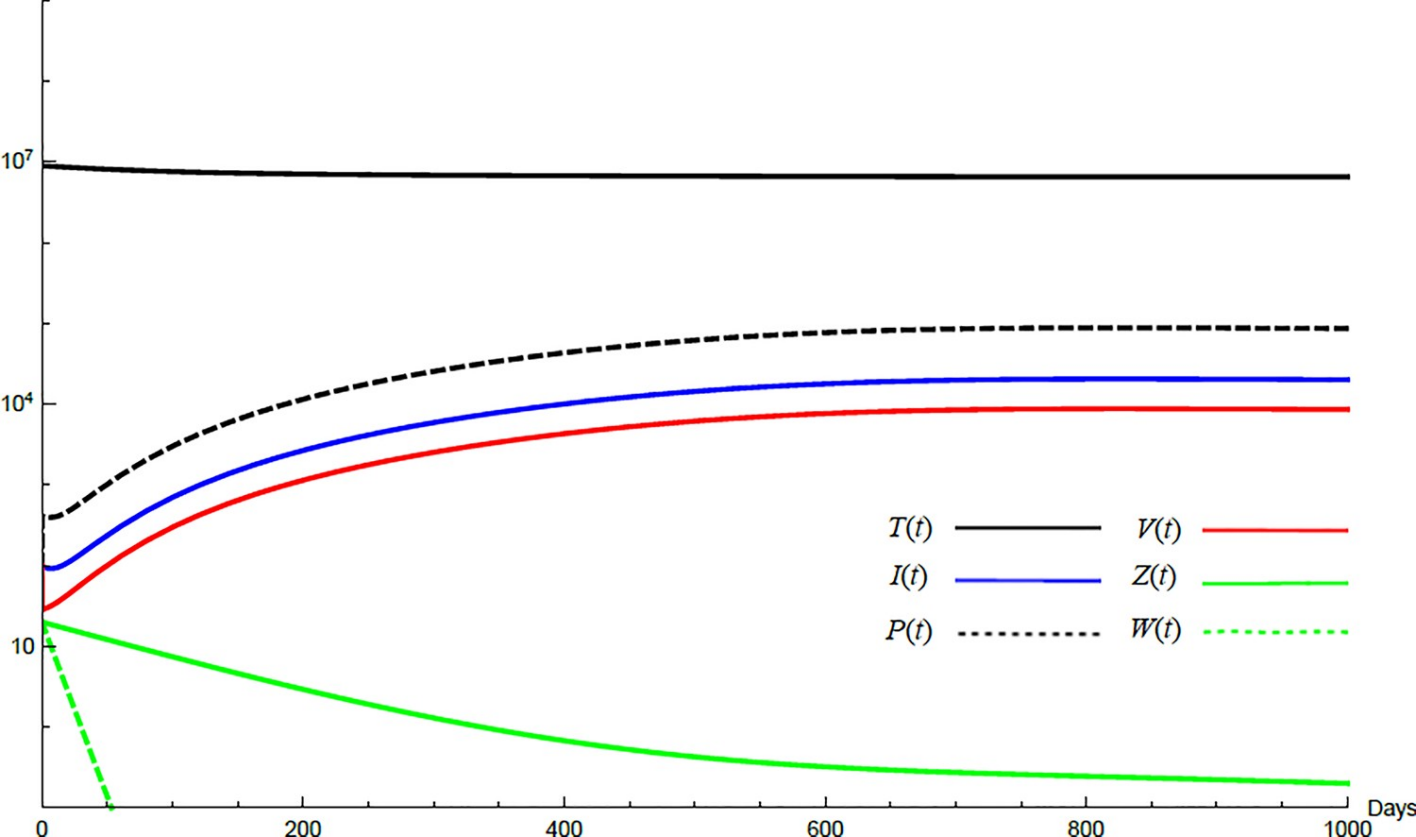
Variation of the variables for no treatment case with *s* = 68225, *c* = 82.3, *R*_0_ = 1.043, *A*_1_ = 1.05, *A*_2_ = 1.049, *A*_3_ = 1.02, *and A*_4_ = 1.07. Hence 1<*R*_0_<*min*(*A*_1_,*A*_2_) and *E*_1_ is stable. *E*_1_ = {6.54×10^6^, 20108, 86603, 8608,0,0}. *T*^0^ = 0.9×10^7^, *I*^0^ = 100, *P*^0^ = 100, *V*^0^ = 100, *Z*^0^ = 20, *W*^0^ = 20.

**Fig 3:**
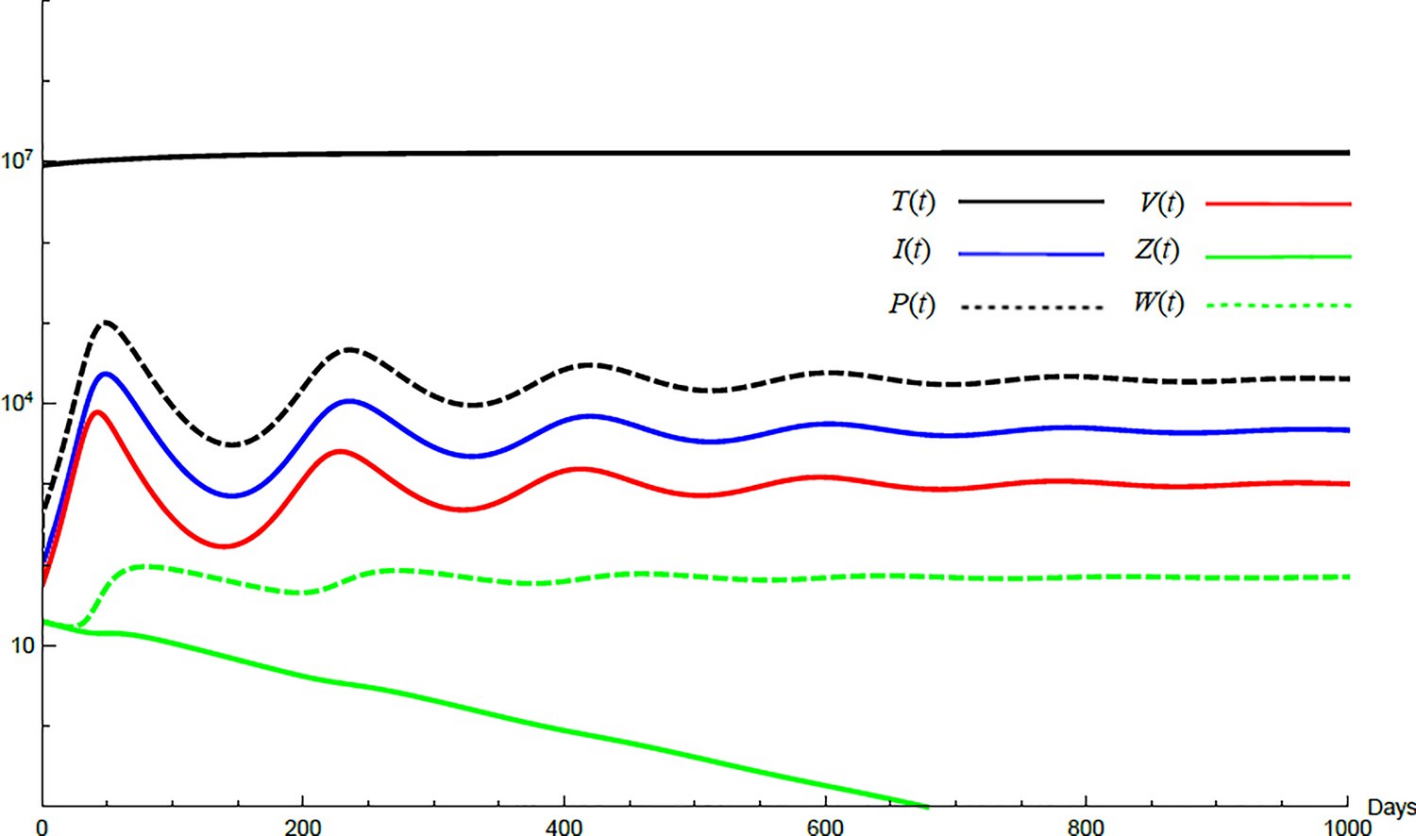
Variation of the variables for no treatment case with *s* = 1.3×10^5^, *c* = 22.3, *R*_0_ = 7.33, *A*_1_ = 1.005, *A*_2_ = 1.18 *A*_3_ = 36.08, *and A*_4_ = 0.204. Hence *A*_1_<*R*_0_<*A*_3_ and *E*_2_ is stable. *E*_2_ = {1.29×10^7^, 4620, 19897, 1000, 0,70}. *T*^0^ = 0.9×10^7^, *I*^0^ = 100, *P*^0^ = 100, *V*^0^ = 100, *Z*^0^ = 20, *W*^0^ = 20.

**Fig 4:**
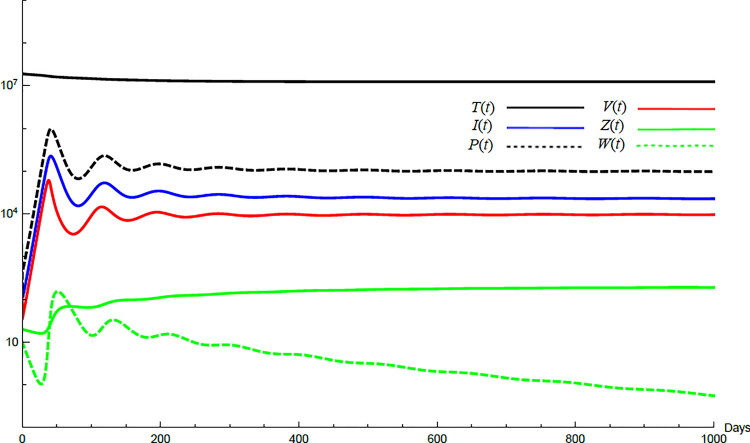
Variation of the variables for no treatment case with *s* = 1.3×10^5^, *c* = 82.3, *R*_0_ = 1.99, *A*_1_ = 1.05, *A*_2_ = 1.049, *A*_3_ = 1.021, a*nd A*_4_ = 2.036. Hence *A*_2_<*R*_0_<*A*_4_ and *E*_3_ is stable. *E*_3_ = {1.24×10^7^, 22727, 98191, 9759, 197,0}. *T*^0^ = 1.9×10^7^, *I*^0^ = 100, *P*^0^ = 100, *V*^0^ = 100, *Z*^0^ = 20, *W*^0^ = 20.

**Fig 5:**
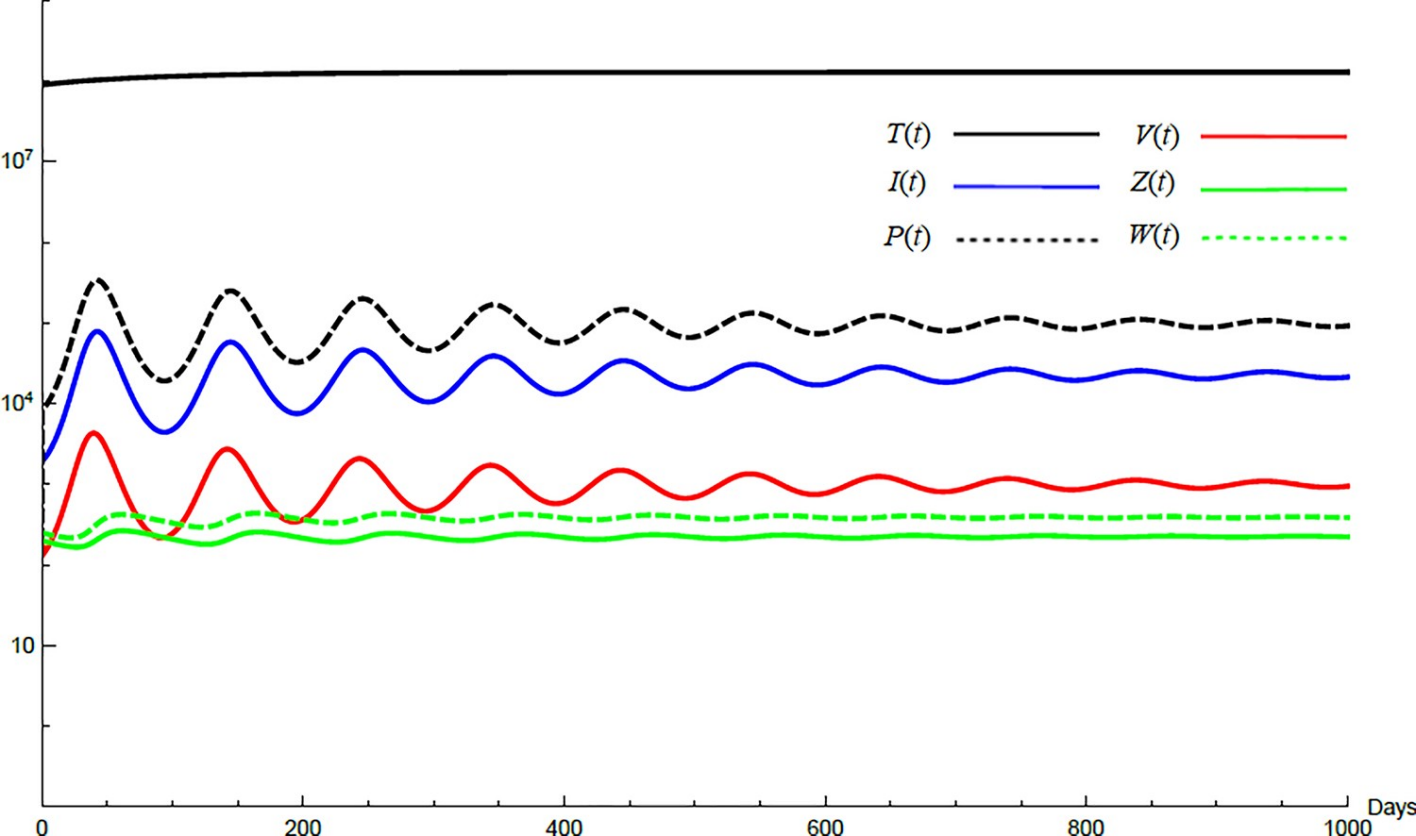
Variation of the variables for no treatment case with *s* = 1.3×10^6^, *c* = 22.3, *R*_0_ = 73.35, *A*_1_ = 1.005, *A*_2_ = 1.18, *A*_3_ = 36.08 *and A*_4_ = 2.036. Hence *R*_0_≥*max*(*A*_3_, *A*_4_) and *E*_4_ is stable. *E*_4_ = {1.29×10^8^, 22727, 98236, 1000, 226, 391}. *T*^0^ = 0.9×10^8^, *I*^0^ = 2000, *P*^0^ = 200, *V*^0^ = 200, *Z*^0^ = 200, *W*^0^ = 250.

To demonstrate the mutual relations between the basic reproduction number, the equilibrium points, and the stability analysis, the form of the proposed model under no treatment represented in Eqs (15)–(20) is simulated first. The simulation demonstrates the variation of all variables *T*(*t*), *I*(*t*), *P*(*t*), *V*(*t*), *Z*(*t*), *W*(*t*) with time. Each figure represents a case with a specific value for the basic reproduction number *R*_0_ which leads to a corresponding equilibrium point that is stable according to theorems 5.1 and 5.2. Figs 1–5 illustrate that the variables converge to that equilibrium point. *T*^0^, *I*^0^, *P*^0^, *V*^0^, *Z*^0^, *and W*^0^ are the initial values of the variables.

**Fig 6:**
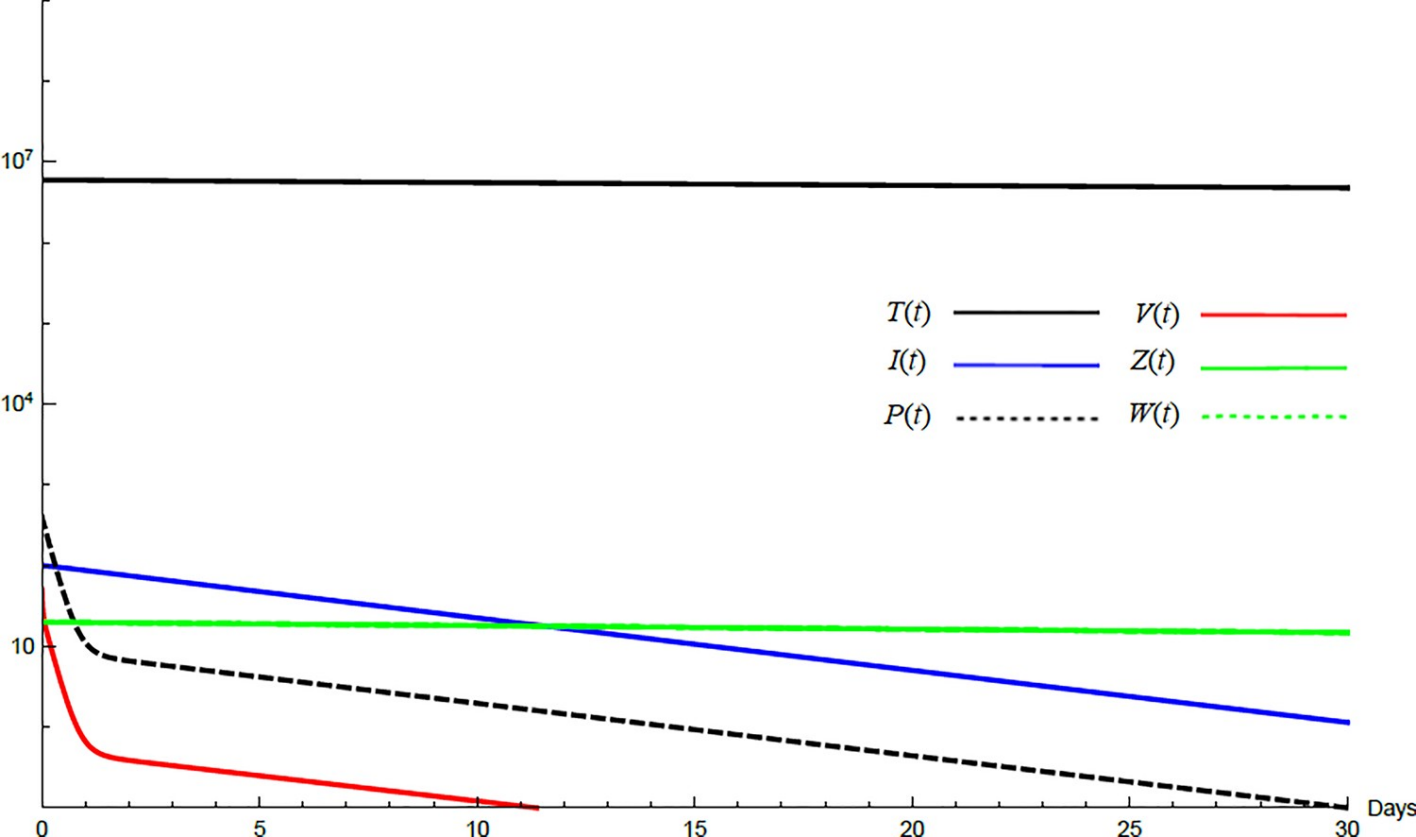
Variation of the variables for medical treatment case with *R*_0_ = 0.733. .

**Fig 7:**
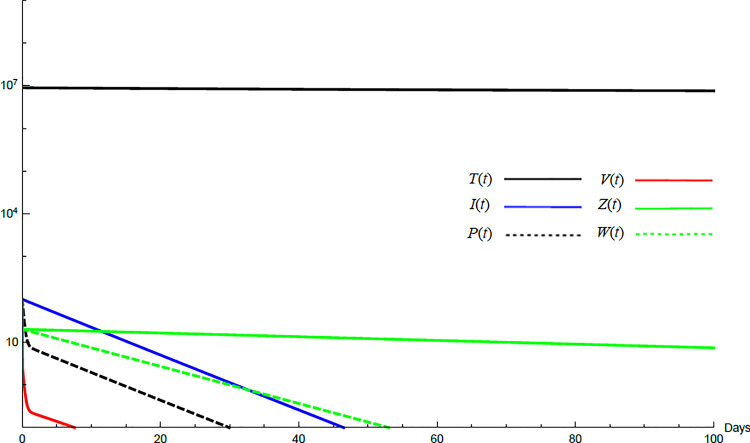
Variation of the variables for medical treatment case with *R*_0_ = 1.043.

**Fig 8:**
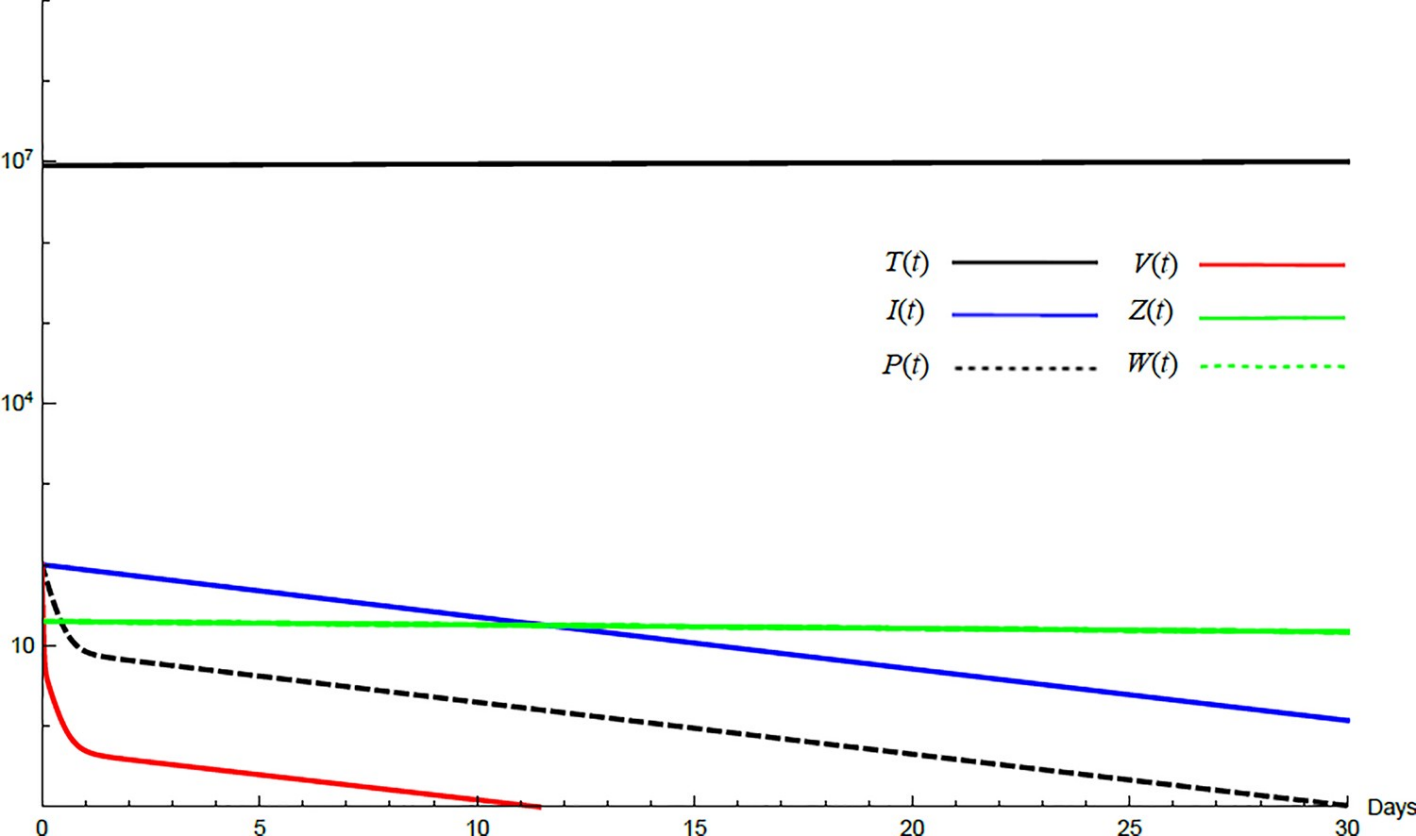
Variation of the variables for medical treatment case with *R*_0_ = 0.733.

**Fig 9:**
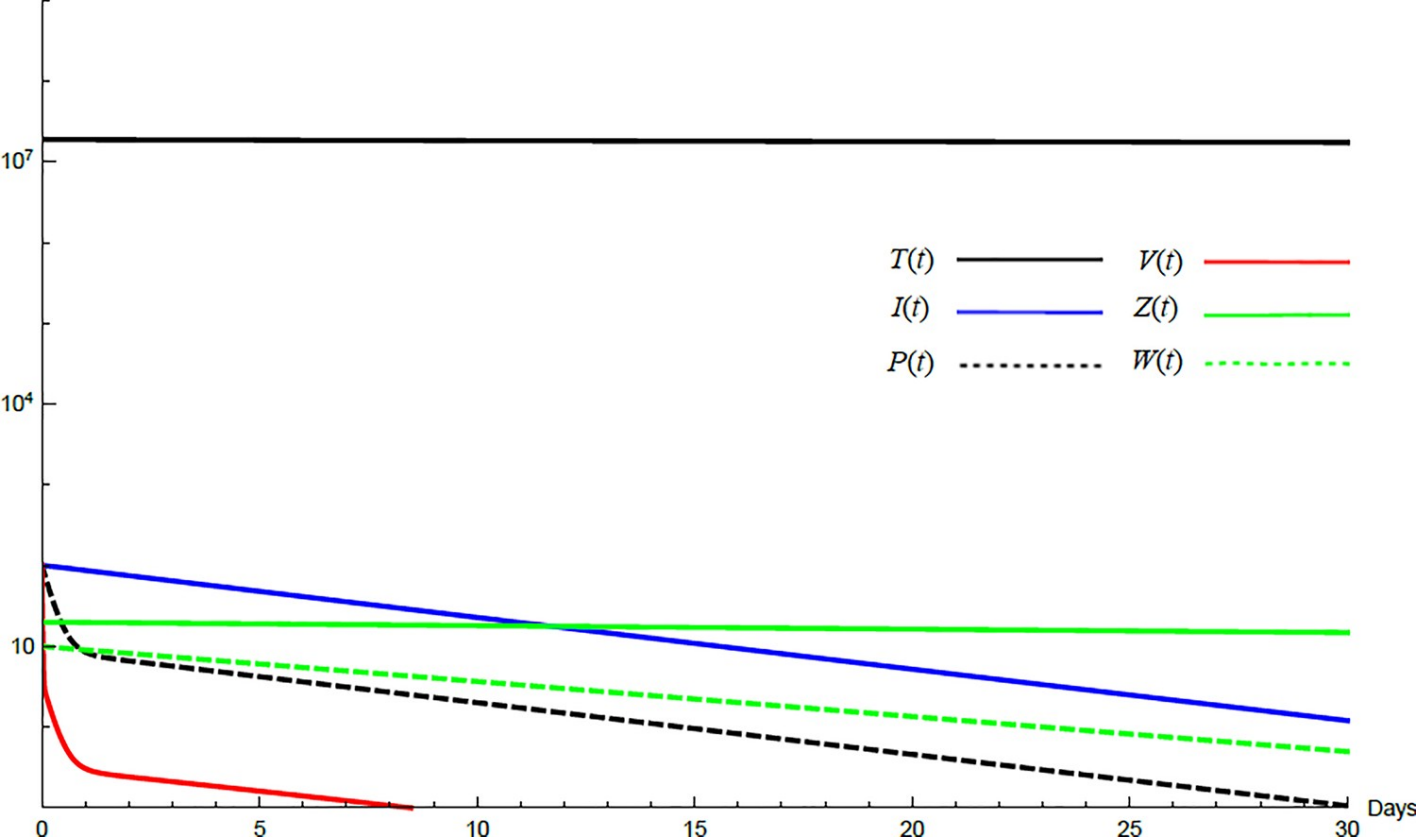
Variation of the variables for medical treatment case with *R*_0_ = 1.99.

**Fig 10:**
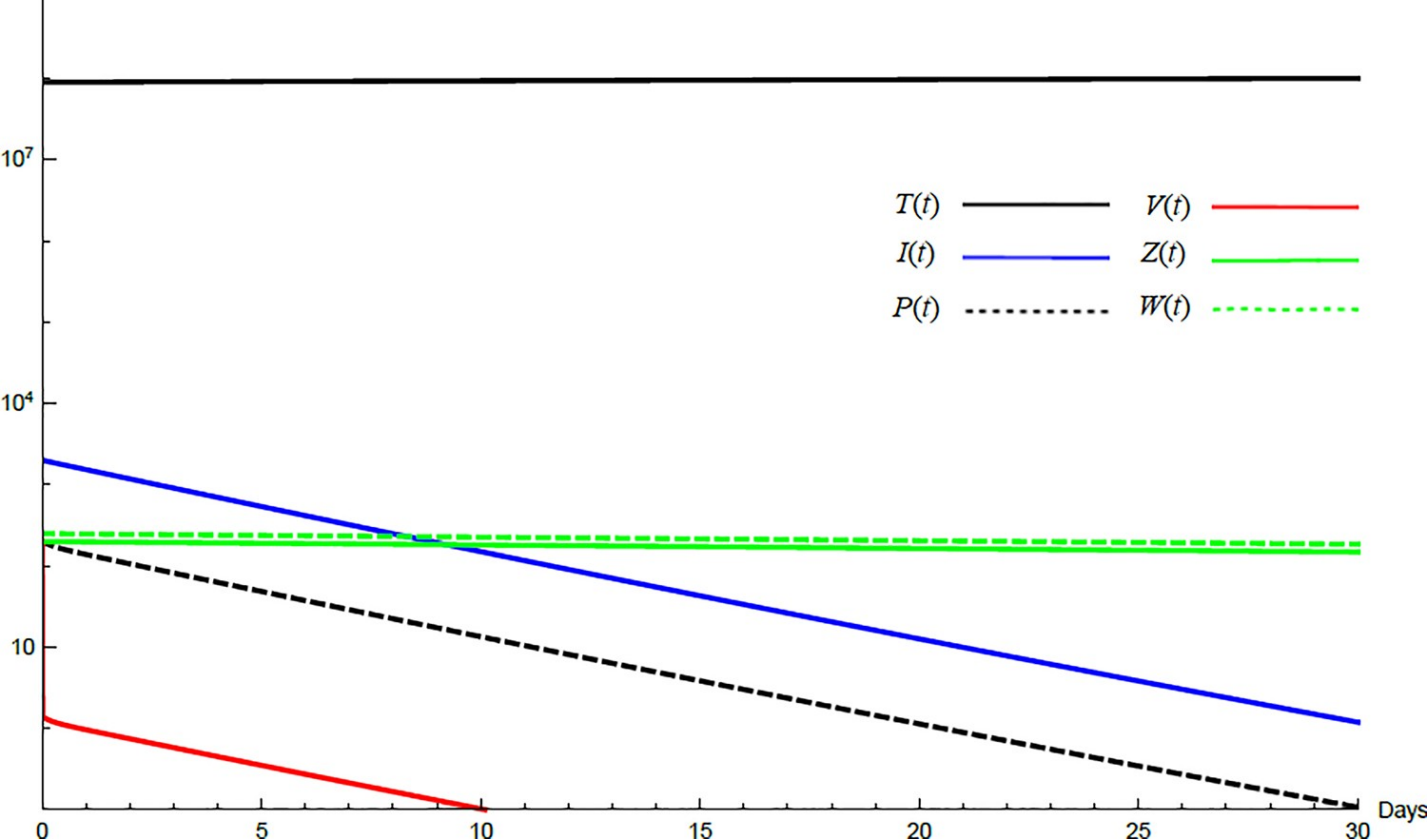
Variation of the variables for medical treatment case with *R*_0_ = 73.35.

To demonstrate the effect of medical treatments, the form of the proposed model under treatment represented in Eqs (9)–(14) is considered now. The same cases considered in the first stage is reconsidered in this stage, however, with medical treatment. Figs 6–10 reveal the effect of medication. Whenever the line for the antibodies is not seen it coincides with the line for CTLs. The simulation proves the practicality and the effectiveness of the proposed model.

**Fig 11:**
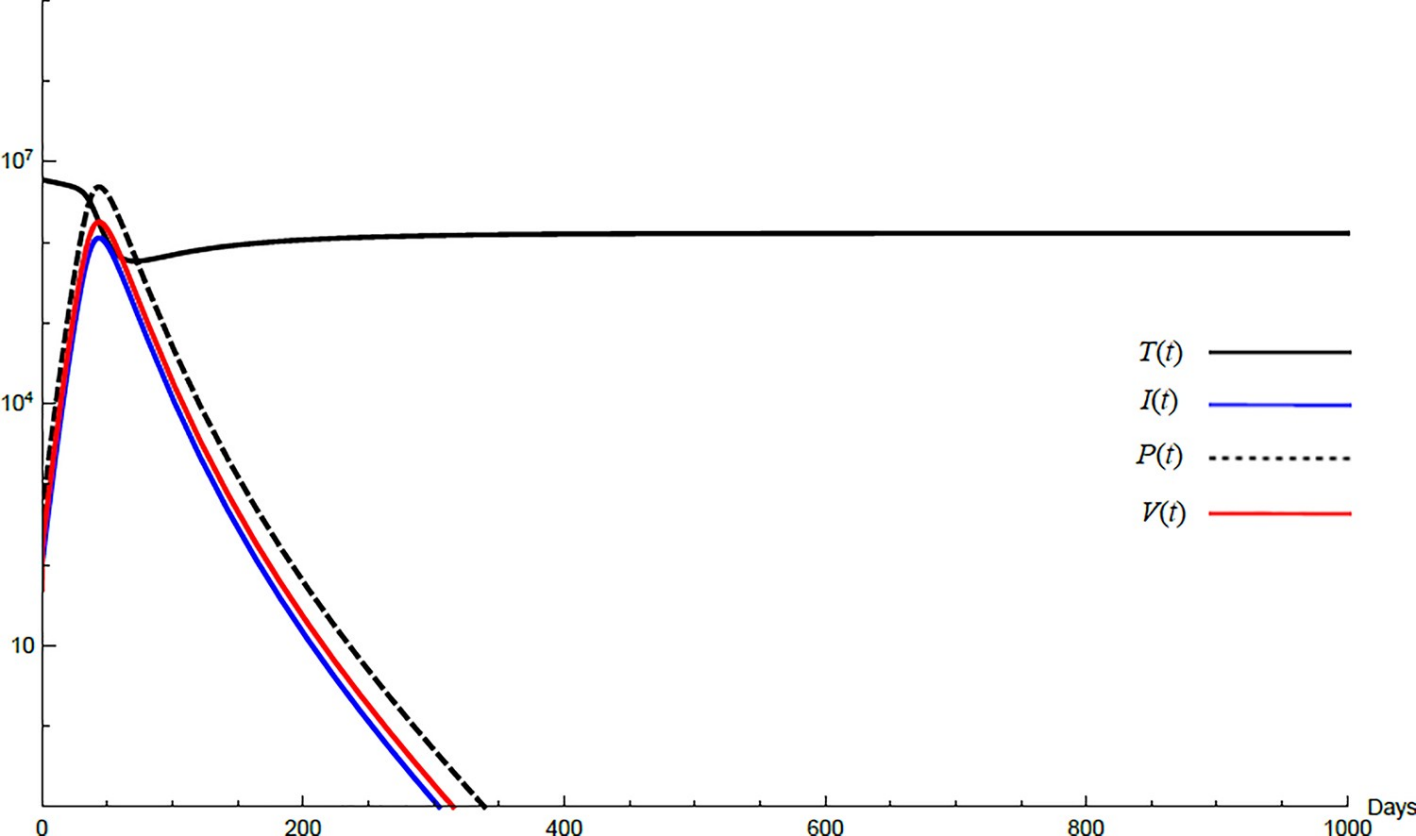
Variation of the variables for the transformed model for no treatment case with *s* = 1.3×10^4^, *c* = 22.3, *R*_0_ = 0.733. Hence *R*_0_<1 and *E*_0_ is stable. *E*_0_ = {1.3×10^6^,0,0,0}. *T*^0^ = 0.6×10^7^, *I*^0^ = 100, *P*^0^ = 400, *V*^0^ = 50.

**Fig 12:**
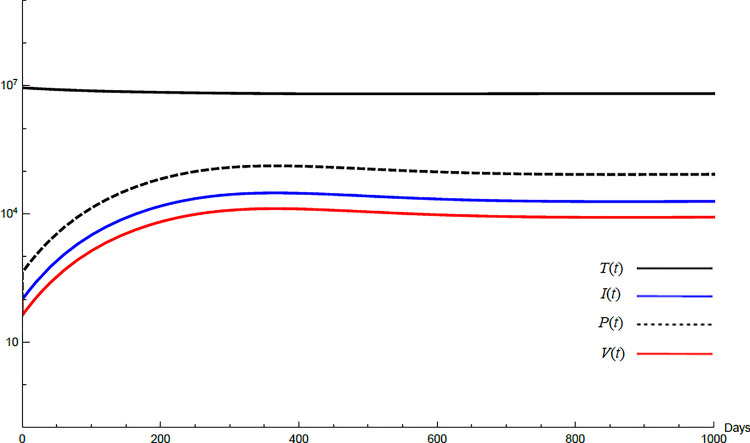
Variation of the variables for the transformed model for no treatment case with *s* = 68225, *c* = 82.3, *R*_0_ = 1.043. Hence *R*_0_>1 and *E*_1_ is stable. *E*_1_ = {6.54×10^6^, 20108, 86603, 8608}. *T*^0^ = 0.9×10^7^,*I*^0^ = 100, *P*^0^ = 100, *V*^0^ = 100.

**Fig 13:**
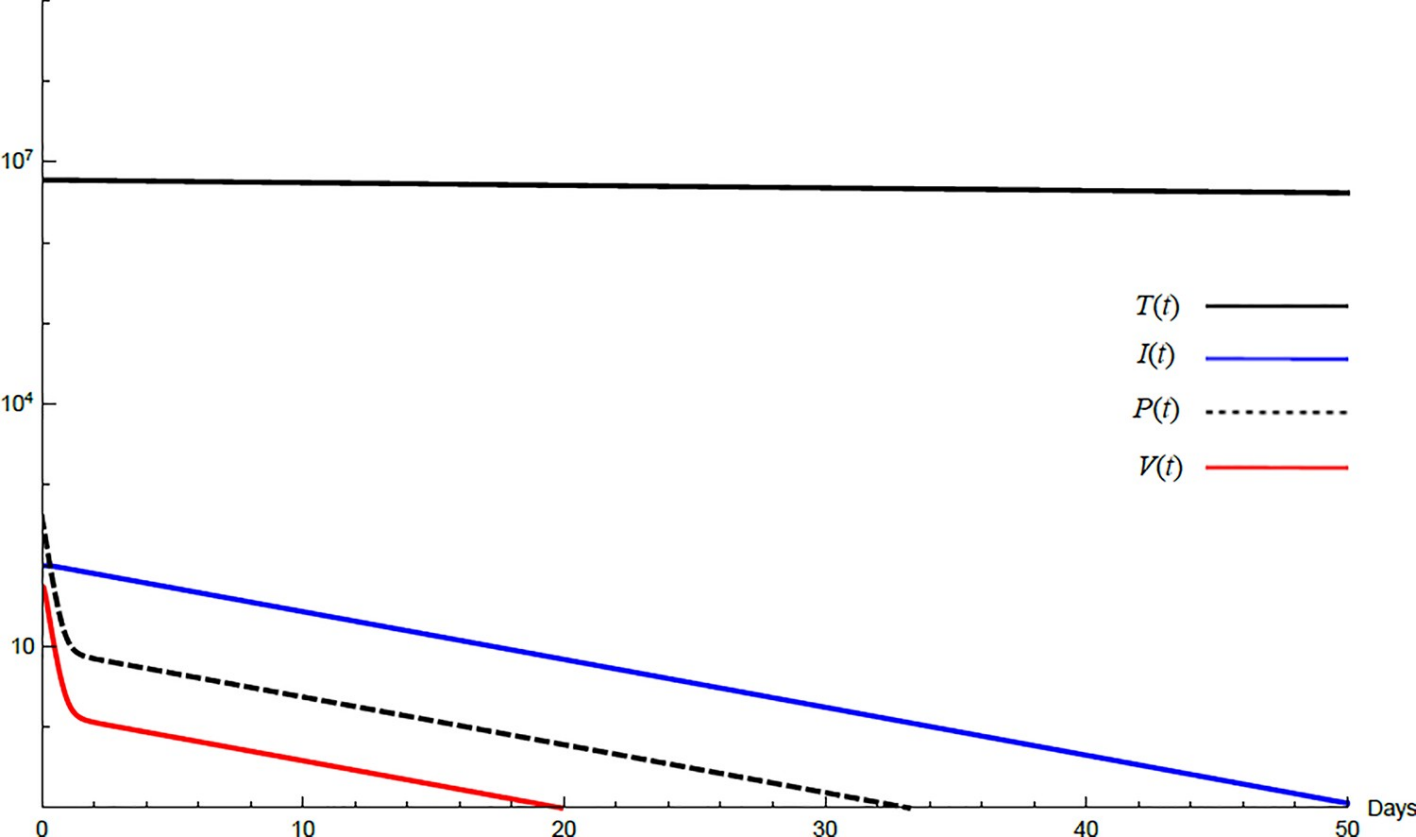
Variation of the variables for transformed model for medical treatment case with *R*_0_ = 0.733.

**Fig 14:**
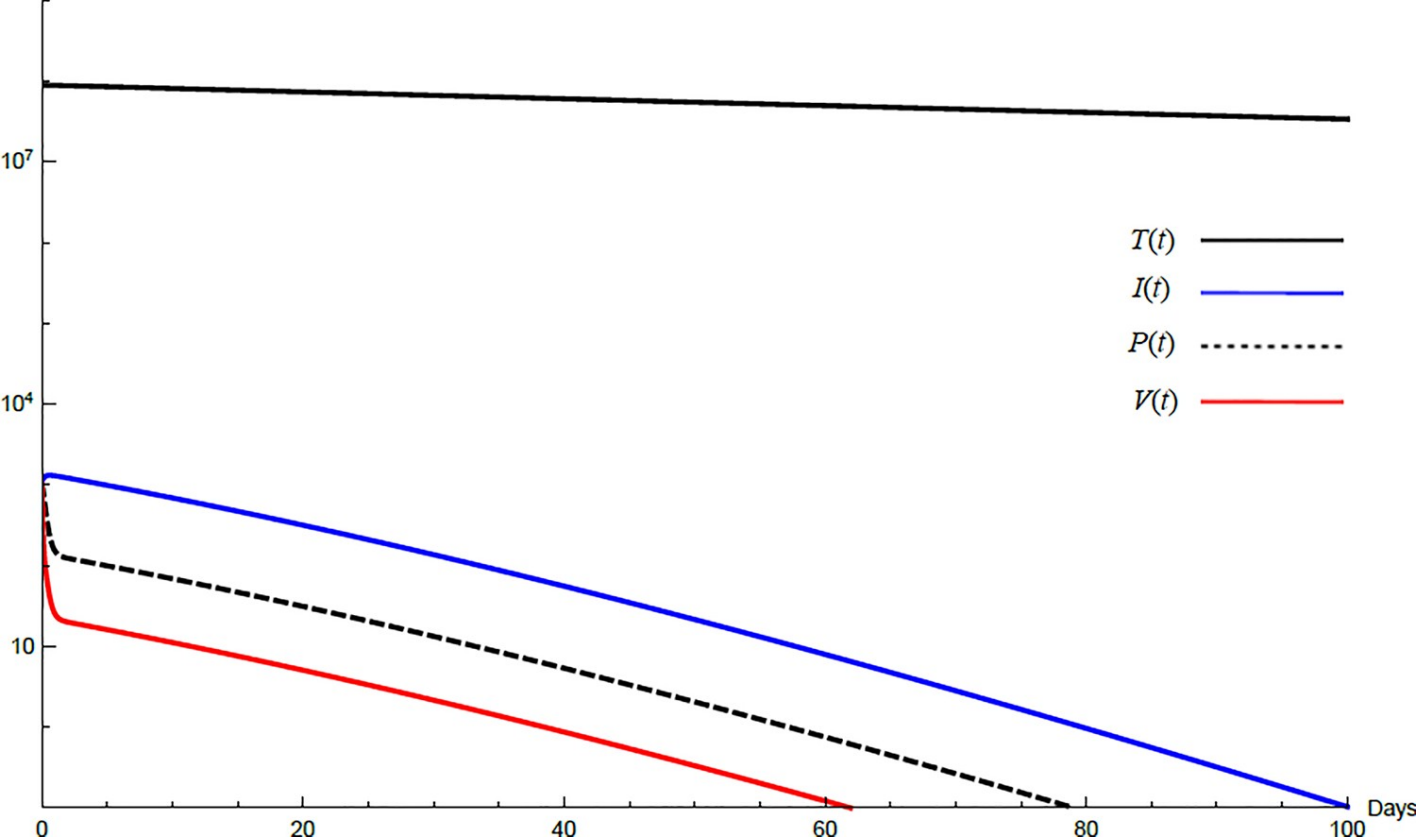
Variation of the variables for the transformed model for medical treatment case with *R*_0_ = 1.043.

For the comparison purpose, the simulation for the transformed model is performed. The transformed model has two equilibrium points only. Hence, two cases are considered, one for the noninfected case and the other for infected case. No treatment illustrations are considered by substituting *ε*_*α*_ = 0, *ε*_*s*_ = 0, *and k* = 1 in Eqs 5–8 and for the medical treatment illustrations, the values in Table 1 for these parameters are used. Comparing Figs 11–14 with Figs 1, 2, 6 and 7 reveal some important outcomes for including the CTLs and antibodies in the model. The comparison shows that CTLs and antibodies smoothen the variation of other variables and reduce the medication time.

## 7. Discussion

It can be notice from Eq (33) that the parameters related to immune system do not affect the basic reproduction number. To explain that, let us go back to the definition of *R*_0_ which implies that we assume that a virus particle enters the system, when *t* = 0 and the system is at the disease-free equilibrium *E*_0_. Accordingly, when *t* = 0, Eqs (15)–(18) implies that the rates of variables *T*,*I*,*P*,*V* have nonzero values since *V*(0) = 1, *T*(0) = *T*^0^. Hence, these variables will vary with time. Opposite to that, when *t* = 0, Eqs (19) and (20) imply that the rates of variables *Z* and *W* have zero values since *Z*(0) = 0, *W*(0) = 0. Hence, *Z and W* will not vary with time i.e., *Z*(*t*) = 0 and *W*(*t*) = 0. Biologically, when one typical viral particle placed in a population consisting only of uninfected cells *T*, ultimately, some of these cells would be infected and became infected *I* cells and consequently *P* intracellular viral RNA cells. However, since initially there are no CTLs and antibodies, they could not be generated. Therefore, the parameters multiplied by *Z and W* would not appear in the formula of *R*_0_.

Including the immune system, raised the number of the equilibrium points to five. The first point is a virus-free equilibrium point, and the second point is an infected point with no immune responses. These two equilibrium points are the same as those obtained using transformed model without the immune system in [34]. The last three equilibrium points give an insight about immune response on the stability of the system and notably they could not be obtained using the transformed model. These three infected equilibrium points are: a point with dominant antibody responses without CTLs, a point with dominant CTL responses without antibodies, and a point with coexistence responses of both CTLs and antibodies, respectively. Both the CTLs and antibodies are stimulated by the virus, so they are in competition. Though role of CTL and antibodies for the resolution of HCV infection is debated in the literature, many medical evidence supports the notion of competition between the two branches of the immune system [42,43]. The infected equilibrium point with dominant antibody means that the antibody response is strong and diminishes virus load to a level that is too low to stimulate the CTL response. The infected equilibrium point with dominant CTLs means that the CTL response is strong and diminishes virus load to a level that is too low to stimulate the antibody response. In these two equilibrium points, the competition between the CTLs and antibodies results in exclusion of one of them. The competition could result in co-existence of both as in the fifth equilibrium point.

Comparing the values of the variables T_*_, I_*_, P_*_, and V_*_ for the equilibrium point with no immune responses *E*_1_ and the equilibrium point with dominant antibody responses *E*_2_, it can be simply proven that *T*_2_≥*T*_1_, *I*_2_≤*I*_1_, *P*_2_≤*P*_1_, and *V*_2_≤*V*_1_ when *E*_2_ exists i.e., *R*_0_≥*A*_1_. The same is applicable to equilibrium point with dominant CTL *E*_3_ responses and equilibrium point with coexistence responses of both CTLs and antibodies *E*_4_. Though, the antibody activation and the CTL activation could not eliminate the viral load, they notably increase the uninfected cells, decrease the infected cells and intracellular viral RNA, and reduce the viral load.

It is worth to notice that theorems 5.1 and 5.2 prove that each equilibrium point has a specific domain of stability. These domains of stability could be overlapped. For example, if *A*_1_≤*A*_2_ and *A*_3_≤*A*_4_, the domains of global stability of *E*_2_ and *E*_3_ will be intersecting except if *A*_3_≤*A*_2_. Hence, a bistable equilibrium could be found, which means the coexistence of two stable equilibrium points. A similar situation had been reported in many biological circumstances, like in multistrain disease dynamics discussed in [36], due to the low capacity for treatment of infective in epidemic models [44], and in investigating bifurcations and stability of an HIV model that incorporates the immune responses [45]. In the presence of bistable equilibria, the solution converges to one of the two stable equilibrium points depending on the initial conditions. Therefore, it is called bistable dominance since the species in the better position originally dominates [36].

Since the proposed model is a multiscale model that incorporate the immune system response, it considers the intracellular viral RNA with the introduction of age-dependency in addition to time-dependency. Hence, the model can explore the dynamics of HCV infection under therapy with DAAs by including both the intracellular viral RNA replication/degradation and the extracellular viral infection with age-dependency in addition to time-dependency. The parameters of the intracellular viral RNA, *P*, appears in both the basic reproduction number and in the coordinates of the equilibrium points. Therefore, the stability of the proposed model is considerably much more difficult to consider and to analyze compared to the corresponding classical model which could not describe the intracellular viral dynamics [27,28,46].

## 8. Conclusions

This work has utilized the transformed multiscale model for HCV in the form of ODE, which is direct and easier to analyze and modify. The immune system, which has a significant role in reducing the virus load, has been incorporated into the multiscale model. The proposed model could represent a valuable tool to comprehend the pathogenesis and controlling treatment of chronic HCV. One of the main advantages of the proposed model over classical multiscale model is its ability to obtain equilibrium points after the cease of medication while in the presence the immune effects. The basic reproduction number of the infection *R*_0_ has a crucial rule in dealing with the stability of the spread of the HCV infection, hence; it has been identified. The parameters related to immune system do not affect the basic reproduction number. The disease-free equilibrium point and the endemic equilibrium points are specified. Conditions for the existence of these points are derived. At any state of the system, only a maximum of five total equilibrium points including the uninfected point can be available. The four infected equilibrium points are: a point with no immune responses, a point with dominant antibody responses without CTLs, a point with dominant CTL responses without antibodies, and a point with coexistence responses of both CTLs and antibodies, respectively. It has been revealed that the four infected equilibrium points are dependent upon the immune system parameters.

Global stability of the equilibrium points has been considered, the Lyapunov principle has been utilized. A new appropriate Lyapunov function has been suggested, hence, sufficient conditions have been derived for the global stability of the five equilibrium points. It has been proven that the uninfected equilibrium point is asymptotically stable if *R*_0_ ≤ 1 and unstable if *R*_0_ > 1. The stability of the four infected equilibrium points depends upon the basic reproduction number and upon the parameters defined by the CTL response and antibody response. Therefore, these parameters play an important role to characterize the stability of the equilibrium points. The activation of antibodies and the activation of CTLs will not eliminate the viral load but they, remarkably, reduce the viral load.

For successful treatment, if *R*_0_>1 the treatment should be directed to improve the body parameters to ensure that *R*_0_≤1 and then the treatment for reducing the virus could be conducted until the state of the body comes to the attracting zone for the stable uninfected point. Consequently, the immune system will lead the state of the body to a stable uninfected state. Otherwise, if an unstable uninfected equilibrium point exists, the virus could not be eradicated even if this uninfected point is approached. Also, a successful treatment ensures that the infected equilibrium points do not exist, so the system would not be attracted by any one of them if it exists.
